# Insights into the High-Pressure Behavior of AWO_4_‑Type Orthotungstates

**DOI:** 10.1021/acs.jpcc.5c07394

**Published:** 2025-12-26

**Authors:** Alfonso Muñoz, Silvana Radescu, Andrés Mujica, Daniel Errandonea

**Affiliations:** † Departamento de Física, MALTA Consolider Team, 16749 Universidad de La Laguna, E-38200 San Cristóbal de La Laguna, Tenerife, Spain; ‡ Departamento de Física, MALTA-Consolider Team, Instituto de Materiales y Nanotecnología, Universidad de La Laguna, E-38200 San Cristóbal de La Laguna, Tenerife, Spain; § Departamento de Física Aplicada - Instituto de Ciencia de Materiales, MALTA Consolider Team, Universidad de Valencia, Edificio de Investigación, C/Dr Moliner 50, 46100 Burjassot, Valencia, Spain

## Abstract

Pressure-induced phase transitions in orthotungstates have resulted in intriguing physical phenomena. The transitions that are observed typically involve significant volume reductions and substantial alterations in the electronic and vibrational characteristics of the materials. In this feature article, we examine the existing knowledge regarding the behavior of AWO_4_ tungstates when subjected to compression. Specifically, we provide a summary of research on their structural and electronic properties, along with several illustrative examples of high-pressure investigations in the relevant compounds. A comprehensive understanding of the high-pressure behavior of AWO_4_ compounds is offered, with a focus on findings that may be pertinent for practical applications. Recent developments and future challenges in the study of orthotungstates under extreme pressure are discussed, along with conclusions that may impact such study. Additionally, some suggestions for topics that could lead to significant breakthroughs will also be presented.

## Graphical abstract

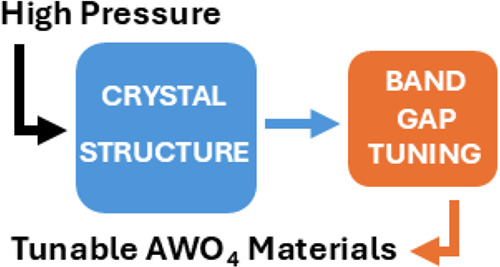

## 1 Introduction

A variety of divalent metal tungstates with the formula AWO_4_ have been the focus of ongoing scientific research. These compounds have been studied for decades because of their multiple technological applications. They have attracted the interest of physicists, chemists, and material scientists because of their applications in photonics and photoelectronics,^1^ their use in detectors at the Large Hadron Collider at CERN,^2^ as laser host materials,^3^ in various optoelectronic devices such as eye-safe Raman lasers,^4^ in photocatalysis,^5^ and due to their importance in earth and planetary sciences.^6^ AWO_4_ orthotungstates form two major families, the scheelite-type group, which includes CaWO_4_, SrWO_4_, BaWO_4_, EuWO_4_, and PbWO_4_, and the wolframite-type group, which includes CdWO_4_, MgWO_4_, ZnWO_4_, FeWO_4_, MnWO_4_, NiWO_4_, and CoWO_4_. However, the two families do not include all AWO_4_ compounds. There are other compounds like CuWO_4_, HgWO_4_, SnWO_4_, AlWO_4_, and CrWO_4_ which have crystal structures different than scheelite and wolframite.

AWO_4_ orthotungstates have attracted attention not only because of their technological applications but also in fundamental research due to their behavior under high-pressure (HP) conditions.^7,8^ High-pressure environments can drive a variety of structural changes and phase transitions in materials, often leading to the formation of previously unknown crystalline phases.^9^ Understanding the behavior of matter under high-pressure conditions is important for designing new materials with desired properties tailored to various applications.^10^ High-pressure research has significantly advanced materials science and geoscience by exploring extreme conditions. Innovative phenomena like pressure-induced metallization^11^ and pressure-driven superconductivity^12^ have transformed the field of materials science.

In the case of AWO_4_ compounds, phase transitions have been reported at pressures as low as 7 GPa.^13^ The observed structural changes have triggered remarkable changes in the electronic, vibrational, and mechanical properties of AWO_4_ orthotungstates.^1^ Consequently, it appears appropriate to provide a comprehensive, systematic overview of the state-of-the-art regarding the HP behavior of AWO_4_ compounds, along with the most significant changes induced by pressure in the physical and chemical properties of these materials.

The article is structured as follows. In the second section, some general structural features of the crystal structure of the different AWO_4_ materials will be presented, including observations and comments regarding their most significant characteristics. In the third section, various aspects concerning pressure-induced phase transitions in these materials are introduced and examined. The two subsequent sections are dedicated to discussing the most pertinent changes in the physicochemical properties of the compounds studied, with a focus on compressibility and electronic properties. The sixth section provides a concise overview of density-functional theory predictions for the few materials belonging to the AWO_4_ family, which have so far been poorly studied. Future directions of research concerning the behavior of AWO_4_ compounds when subjected to compression, which warrant further investigation, will be addressed toward the end of the manuscript.

## 2 Crystal Structures

AWO_4_ tungstates typically crystallize in a scheelite-type tetragonal structure^14^ characterized by the space group *I*4_1_/*a* when the ionic radius of the cation A exceeds 1.0 Å (A = Ba, Ca, Sr, Eu, Pb), featuring a tetrahedral coordination of tungsten. Alternatively, for A cations with an ionic radius of less than 1.0 Å (A = Cd, Co, Fe, Mg, Mn, Ni, Zn), they usually adopt a wolframite-type monoclinic structure^15^ described by space group *P*2/*c*, with an octahedral coordination of tungsten. Both types of compounds are routinely synthesized in laboratories and growth as single crystals, but they can also be found as minerals in Nature. The two crystal structures are schematically represented in Figure 1.

**Figure 1:**
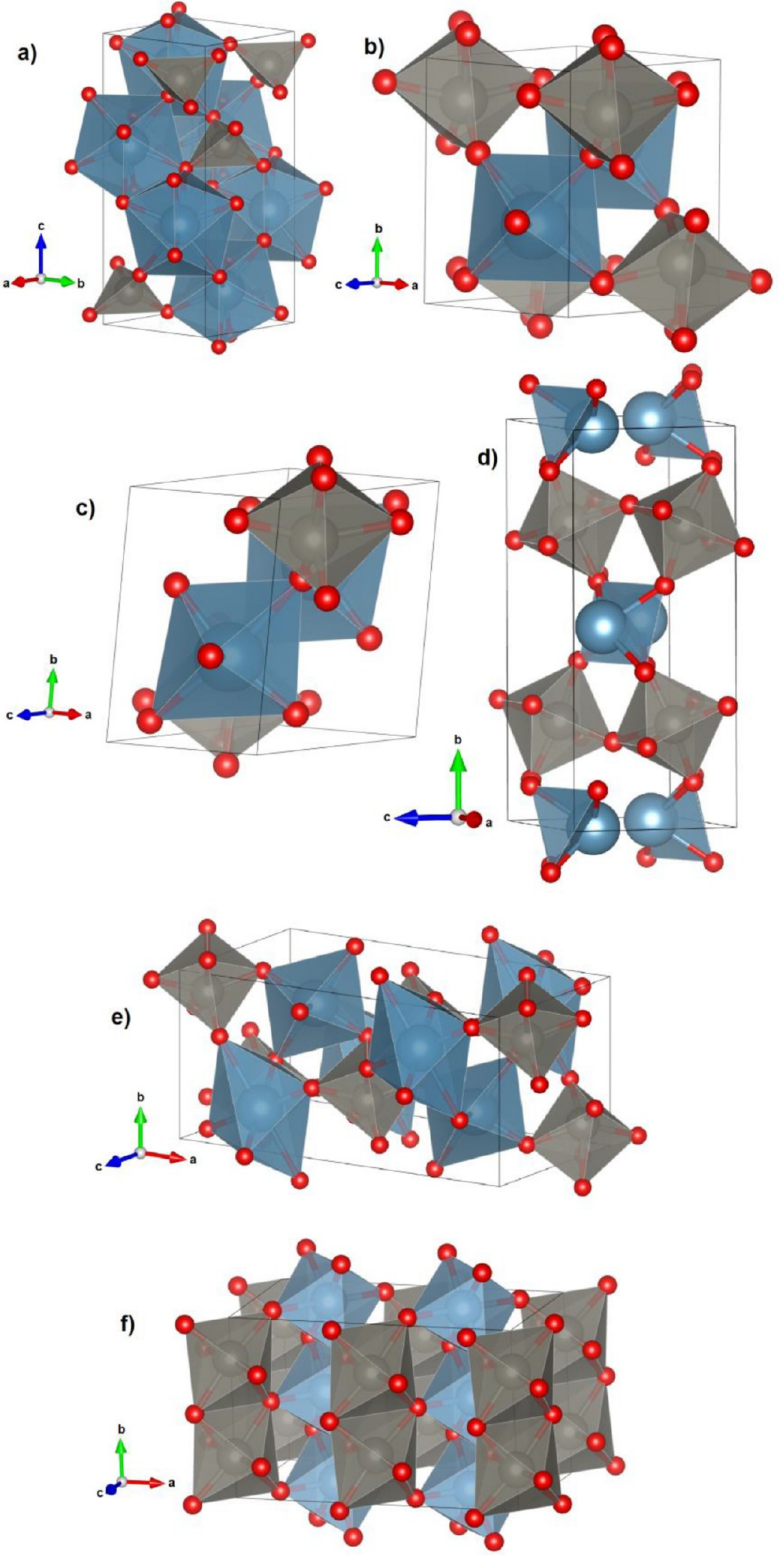
Crystal structure of (a) scheelite, (b) wolframite, (c) CuWO_4_, (d) SnWO_4_, (e) HgWO_4_, and (f) AlWO_4_ (CrWO_4_). Coordination polyhedral of A cations and W atoms are shown in blue and gray, respectively. Oxygen atoms are represented in red.

Scheelite is a calcium tungstate mineral with the chemical formula CaWO_4_. Scheelite-type AMO_4_ compounds are isostructural to CaWO_4_. They are prevalent binary oxides found in both natural and synthetic systems.^16^ Scheelite-type tungstates are commonly found in skarn-type deposits. The scheelite structure, described by space group *I*4_1_/*a*, is highly versatile and can accommodate A cations with valence +1, +2, +3, and +4 in conjunction with M cations with valence +7, +6, +5, and +4, respectively. The crystal structure is characterized by eight-coordinated A cations and tetrahedrally coordinated B cations. In AWO_4_ scheelites, the primary polyhedra are the coordination tetrahedron surrounding W and the bisdisphenoids of coordination surrounding A atoms. The structure of scheelite can be described as two interpenetrating diamond networks, one for the A cations and the other for the W atoms. As shown in Figure 1a, the structure is formed by a network of interconnected edge sharing AO_8_ dodecahedra. Additionally, these units also share corners with adjacent WO_4_ regular tetrahedra. These tetrahedra remain isolated from each other. From this point forward, we will refer to all AWO_4_ compounds exhibiting this crystal structure as scheelites.

Wolframite is an iron manganese tungstate mineral with the chemical formula (Fe,Mn)­WO_4_. Wolframite-type tungstates are prevalent in vein-type deposits. The name wolframite is normally used to denote the family of isomorphic compounds. The wolframite structure is monoclinic, belonging to the space group *P* 2/*c*,^17^ and is shared by various AWO_4_ tungstates, ANbO_4_ niobates, ATaO_4_ tantalates, and AMoO_4_ molybdates.^18^ Hereafter, we use the name wolframite to describe all AWO_4_ compounds with this crystal structure. The structure of wolframite is illustrated in Figure 1b. It is formed on a distorted hexagonal close packing of O atoms, with A and W atoms each occupying one-fourth of the octahedral interstices. The arrangement of AO_6_ and WO_6_ octahedra is depicted in Figure 1b. Like octahedra are connected by edges forming alternating infinite zigzag chains running along the [001] direction which confers the structure with a layer-like AOWO configuration in the [100] direction. On the other hand, different octahedra are connected by corners, creating a complex network.

CuWO_4_ is a version with reduced symmetry of wolframite. It has a triclinic structure described by space group *P*1̅.^19^ The structure, shown in Figure 1c, is related to that of wolframite, but with a distortion due to the strong Jahn–Teller distortion of the CuO_6_ octahedra which makes the copper atoms to have a more irregular coordination compared to the divalent A metals in wolframites. The distortion of the CuO_6_ octahedra reduces the symmetry of the crystal structure. This is primarily accomplished through a shear that runs parallel to the [010] direction along each copper plane. This shear causes the oxygen layers surrounding the copper atoms to become slightly misaligned with respect to one another. The resulting displacement disrupts the 2-fold symmetry and is directly observable in the deviation of the angle α and γ from 90°. The structure is shared by CuMoO_4_ and other molybdates.^20^

SnWO_4_ has a distinctive crystal structure. This fact is related to the lone-pair stereochemical activity of the Sn^2+^ s^2^ valence electrons, which are not shared with another atom. This causes a crystallographic distortion, which gives unique characteristics to the crystal structure of SnWO_4_.^21^ In the most stable structure of stannous tungstate, known as α-SnWO_4_, Sn atoms are 4-fold coordinated to oxygen atoms in a trigonal bipyramidal configuration with a Sn atom in the vertex of the pyramid (see Figure 1d). There are additionally two oxygen atoms in the direction opposite to the base of the bipyramid, which favors that under compression the coordination of Sn becomes 4 + 2. The crystal structure is orthorhombic and is described by space group *Pnna*.^21^ The tungsten atoms are coordinated in an octahedral arrangement by oxygen atoms, with the WO_6_ octahedra interconnected at four corners. Consequently, the tungsten and oxygen atoms create sheets of [WO_4_]^2–^ polyanions, which are held together by Sn atoms exhibiting a formal valency of +2. SnWO_4_ has a second metastable polymorph, β-SnWO_4_, which is cubic and is described by space group *P*2_1_3.^22^

The structure of HgWO_4_ is isomorphic to that of HgMoO_4_ and is described by the monoclinic space group *C*2/*c*. As shown in Figure 1e, the crystal structure is formed by zigzag chains of edge-sharing WO_6_ octahedra that stack parallel to the [001] direction. The oxygen atoms form layers in a nearly cubic close-packing arrangement, but with adjacent layers being slightly offset from one another.^23^ Consequently, the octahedral voids that house the mercury atoms exhibit significant distortion.^23^ The Hg atoms are coordinated to six oxygen atoms in a highly distorted octahedral configuration. The HgO_6_ octahedral units also create zigzag chains that extend along [001]. The structure of HgWO_4_ bears a close resemblance to the wolframite structure due to the interconnected nature of the polyhedra. Nevertheless, the coordination polyhedron surrounding the mercury atom distinguishes the HgWO_4_ structure from wolframite. It has second neighbor oxygen atoms close to the first sphere of coordination. Therefore, the coordination can be considered as 6 + 2.

AlWO_4_ and CrWO_4_ are two compounds that share a common crystal structure,^24,25^ which is shown in Figure 1f. The structure is monoclinic and is described by space group *C*2/*m*. It is related to the layered, two-dimensional crystal structure of CrPS_4_. Both materials consist of puckered, hexagonally close-packed oxygen layers with Al^2+^ (Cr^2^^+^) and W^6+^ ions in octahedral coordination. The structure is characterized by CrO_6_ octahedra that share corners with WO_6_ octahedra and edges with other CrO_6_ octahedra. For CrWO_4_, an orthorhombic structure described by space group *F*222 has also been reported.^26^

## 3 Pressure-Induced Transitions

Several phase transitions have been discovered under high-pressure conditions in scheelite-type tungstates. X-ray diffraction studies have determined that CaWO_4_ undergoes a pressure-induced phase transition from its tetragonal scheelite structure to a monoclinic structure, specifically the fergusonite-type structure (*I*2/*a*), at around 11 GPa.^27–29^ As illustrated in Figure S1 in the Supporting Information (SI), the transition is characterized in XRD patterns by the splitting of several peaks and the appearance of an extra peak at low angles. This transition is reversible upon pressure release. This transition is often described as a ferroelastic phase transformation^30^ and it is induced by polyhedral tilting. This phase transition is triggered by the softening of one of the translational Raman modes of the scheelite phase that involves a rotation of the WO_4_ tetrahedra.^31^

A characteristic of the scheelite-fergusonite transition is that the low-pressure structure shows a certain degeneration of the vibrational modes, which disappears once the phase transition to the low-symmetry structure is accomplished. A splitting of several Raman modes at the transition pressure has been reported.^32^ The scheelite-fergusonite transition shows no major change in cation coordination, except for a distortion of the WO_4_ tetrahedron, which becomes slightly irregular, and subtle modifications in the coordination of those cations with the larger coordination number, i.e., the divalent cations. They also occur in a sudden and reversible manner, leaving the crystal lattice undamaged during the transformation and with reduced volume changes.

Powder XRD experiments performed up to 28 GPa confirmed the stability of the fergusonite phase up to this pressure in CaWO_4_. At higher pressures, a second phase transition has been observed in Raman experiments around 33 GPa.^33^ The second HP phase remains stable up to 46.3 GPa, the maximum pressure achieved in the Raman study. Density-functional theory (DFT) calculations suggest that the second transition is to an orthorhombic structure described by space group *Cmca*.^33^ Such transition involves an increase of the coordination number of both Ca and W atoms and a 10% volume collapse. Additionally, other studies have reported amorphization at higher pressures, around 40 GPa.^34^ It has been argued that this could be favored by nonhydrostatic conditions in the pressure chamber. In another study, it was shown that a scheelite-wolframite transition was induced under highly nonhydrostatic conditions.^34^ This transition is related to a shear displacement of the oxygen atoms that are second neighbors of tungsten. It has been also reported that a novel high-pressure phase can be obtained by heating amorphous CaWO_4_ at 45 GPa to 477 K. This phase was quenched as a metastable phase at room temperature-^35^ Its crystal structure is monoclinic (space group *C*2/*m*) and was described as isostructural to α-MnMoO_4_. In this structure both, Ca and W atoms have a distorted octahedral coordination. In Figure 2 we summarize all the results described above and compare the HP behavior of CaWO_4_ with results from other scheelite-type tungstates.

**Figure 2:**
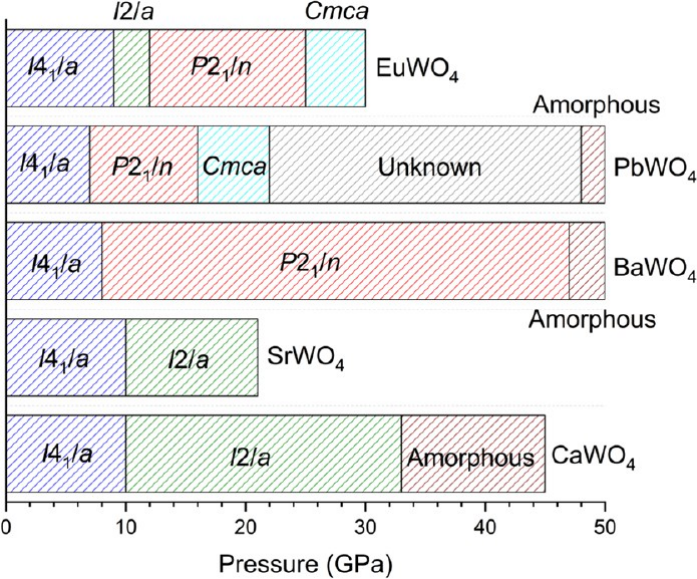
Summary of the reported high-pressure phase transitions in scheelite-type AWO_4_ tungstates. The crystal structures are identified by the corresponding space group.

SrWO_4_ was studied by XRD^28,36^ and by Raman spectroscopy^37^ up to 21 GPa. In this pressure range, the behavior of SrWO_4_ is analogous to that of CaWO_4_. The pressure-induced phase transition in SrWO_4_ from the scheelite structure to the fergusonite structure occurs around 10–12 GPa, depending on the experiment. Further transitions are theoretically predicted to occur at higher pressures, but beyond the pressure range covered by the experiments. The scheelite-fergusonite transition has also been observed in EuWO_4_ at 8 GPa.^38^ In this compound, experiments were performed only up to 12 GPa, and the fergusonite phase was observed at this pressure. Further transitions were predicted at higher pressure by means of DFT calculations.^39^ The predicted phases share structures with the HP phases of PbWO_4_ and BaWO_4_, which will be discussed in the next paragraph, but they need to be confirmed by future experiments. Interestingly, there is one study reporting the synthesis of RaWO_4_ and its characterization using X-ray diffraction.^40^ The report showed that RaWO_4_ crystallizes at ambient conditions in the scheelite structure. However, this compound has not been studied under compression, probably due to the radioactivity and toxicity of radium.

BaWO_4_ and PbWO_4_ have a HP behavior that differs from that of CaWO_4_. In these two compounds, a monoclinic structure known as BaWO_4_–II or PbWO_4_–III, respectively, and described by space group *P*2_1_/*n*, has been synthesized combining high-pressure and high-temperature.^41,42^ The structure has no direct resemblance either to the scheelite- or to the wolframite-type structure. It consists of densely packed layers of WO_6_ octahedra, which are connected by edge- and corner-sharing; barium (lead) atoms are located between them. The coordination number of the barium (lead) atoms has increased to ten.

BaWO_4_ has been studied by powder XRD using diamond-anvil cells.^43–13^ It has also been studied by Raman spectroscopy.^13,45^ In a XRD study, under nonhydrostatic conditions^43^ it was found that the scheelite structure transforms into the fergusonite structure at 7 GPa. A second transition to a disordered structure was found at 14 GPa.^43^ A second study combining XRD, X-ray absorption measurements, and DFT, confirmed the scheelite-fergusonite transition and assigned the second HP phase to the BaWO_4_–II structure.^44^ This study showed that the fergusonite structure is, however, metastable and can only occur if the transition to the BaWO_4_–II phase is kinetically inhibited. This study also indicates that BaWO_4_ becomes amorphous beyond 47 GPa. These conclusions were supported by Raman measurements.^45^ All the experiments observing the scheelite-fergusonite-BaWO_4_–II sequence were performed under conditions that become nonhydrostatic at pressures close to the onset of the first phase transition. This fact has been found to influence the results. This was demonstrated by XRD and Raman experiments under hydrostatic conditions.^13^ In these experiments it was found that BaWO_4_ transforms directly from its low-pressure tetragonal structure into the much denser BaWO_4_–II structure at 8 GPa at room temperature. In addition, a highly nonhydrostatic experiment, without using any pressure-transmitting medium, performed by the same authors,^13^ has resulted in a phase transition to a structure completely different than fergusonite and BaWO_4_–II.

Scheelite-type PbWO_4_ (known as stolzite) has been studied under high-pressure by powder XRD and Raman spectroscopy.^44,46–48^ In contrast with the other AWO_4_ scheelites, PbWO_4_ is dimorphic. In addition to the scheelite-type structure, it has a second polymorph known as raspite.^49^ The structure of this second polymorph is monoclinic, described with space group *P*2_1_/*a*. Raspite is a metastable phase under normal conditions of pressure and temperature and is typically found in natural crystals. It has a one-dimensional chain-like structure formed by edge-sharing WO_6_ octahedra, and Pb ions are coordinated to seven oxygen atoms. This structure was proposed as one of the possible high-pressure phases of PbWO_4_ but has not been observed in HP experiments.^44,46^ Under high pressure, PbWO_4_ undergoes several structural phase transitions. One of the HP phases is orthorhombic, with the same space group as that reported for CaWO_4_ (space group *Cmca*); the other has not been unequivocally determined. Its structural sequence is analogous to that of BaWO_4_. Initially, at around 6.8 GPa, it undergoes a transition from the scheelite structure to the monoclinic structure PbWO_4_–III. Further transitions to other phases occur at higher pressures.^47^ At 47.7 GPa, PbWO_4_ undergoes pressure-induced amorphization. The amorphization occurs at a similar pressure than in BaWO_4_. This phenomenon might be intrinsic, due to the frustration of a solid–solid phase transition, due to the existence of a large energy barrier precluding the occurrence of the transition or might be caused by nonhydrostatic effects. The final explanation for amorphization would require the performance of additional studies.

AWO_4_ wolframites are much more stable than scheelite under compression. This is partly because the smaller atomic radii of the divalent cations of wolframites have stronger bonds than the larger divalent cations of scheelites. This also means that wolframites are less deformable under stress and support higher pressures than scheelites.^18^ CoWO_4_ has been studied by XRD and Raman spectroscopy up to 10 GPa, and the wolframite structure was retained.^50^ FeWO_4_ was studied by XRD^8^ and neutron diffraction^51^ up to 20 GPa, and no phase transition was found. This is exemplified in Figure S2 in the SI, where we present a selection of XRD patterns measured at different pressures. The only difference between the XRD patterns measured at the lowest and highest pressure is the separation between pairs of Bragg peaks that are close to each other at ambient pressure. For instance, the two peaks that are near 6° in Figure S2 (SI). This is a consequence of the anisotropic compressibility of wolframite.

HP studies in MgWO_4_, ZnWO_4_, NiWO_4_, CdWO_4_, and MnWO_4_ provided evidence that under compression, most wolframites undergo a phase transformation to a different polymorph close to 20 GPa.^52–7^ MgWO_4_, ZnWO_4_, and MnWO_4_ apparently transform into a lower symmetry triclinic structure,^52,53^ which has similitudes to that of CuWO_4_, with space group *P*1̅. In contrast, CdWO_4_ under pressure, increases its space-group symmetry,^54^ introducing a screw axis, changing the space group to *P*2_1_/*c* and doubling the unit cell. A similar transition, involving a doubling of the unit cell, but preserving space group *P*2/*c*, has been recently proposed to take place in NiWO_4_ at 27 GPa,^7^ with the HP phase remaining stable up to 50 GPa.

The solution of the HP phase has been approached unsuccessfully with powder X-ray diffraction^52^ in ZnWO_4_ and MgWO_4_ and with single crystal X-ray diffraction in MnWO_4_.^53^ Nevertheless, through a meticulous indexing of the observed reflections, the investigation into the systematic extinctions of the HP phase, along with the count of active Raman modes identified in the HP phase, suggests that a triclinic structure is the most probable postwolframite structure. In the case of MnWO_4_, despite the small volume change of only 1% that occurs in the phase transition, the crystal dramatically deteriorates at the transition with the appearance of more than two triclinic HP domains during the phase transition, coexisting with the monoclinic low-pressure phase. This fact unfortunately prevents a correct integration of the reflection intensities and therefore an accurate determination of the atomic positions in the HP phase. Note that the single-crystal XRD experiments in MnWO_4_ were carried out under controlled hydrostatic conditions. Then, the phase coexistence observed is likely inherent to the properties of MnWO_4_ and not caused by nonhydrostatic effects. In contrast with scheelite, the phase transitions in wolframites do not involve substantial changes in the coordination of W and the divalent cation. The only exception is CdWO_4_ in which in the HP phase Cd and W atoms are 7-fold coordinated. Another important observation is that amorphization has not been observed in wolframites up to 50 GPa.

The HP behavior of CuWO_4_ has been investigated up to 33.9 GPa by means of high-pressure single-crystal X-ray diffraction and extended X-ray absorption fine structure.^19^ Beyond 9 GPa, a phase transition takes place. The transition is from the triclinic (*P*1̅) structure to a monoclinic (*P*2/*c*) structure isotypic to wolframite. The transition implies abrupt changes of CuO_6_ and WO_6_ octahedra, but no coordination change. The Jahn–Teller distortion of the CuO_6_ octahedra plays a key role in the mechanism of the phase transition as well as the changes in the HP behavior of the Cu–O bonds for the triclinic and monoclinic phases of CuWO_4_.^55^ The elongation of the CuO_6_ octahedra due to this effect is influenced by pressure, and the high-pressure phase transition is related to changes in this distortion. A second phase transition was detected at 22.5 GPa.^19^ Both phase transitions are reversible upon pressure release, with the material reverting to its original triclinic structure.

Early theoretical calculations predicted that at high pressures, HgWO_4_ would transform into either a BaWO_4_–II-type or an orthorhombic phase described by space group *Cmca*, both of which are known high-pressure phases for other AWO_4_ compounds.^56^ However, experimental studies using X-ray diffraction and Raman scattering up to 16 and 25 GPa, respectively, found that the monoclinic *C*2/*c* structure of HgWO_4_ remains stable within these pressure ranges.^56^ Ab initio calculations suggest that at higher pressures, the wolframite structure becomes more stable than the HgWO_4_-type structure. The two structures are closely related, with the wolframite structure being a more symmetric version of the HgWO_4_-type structure. Understanding the high-pressure behavior of HgWO_4_ is relevant due to its potential applications in areas like luminescence, detectors, and calorimeters in high-energy experiments. Further research is needed to fully characterize the HP behavior of HgWO_4_.

Studies on α-SnWO_4_ under high pressure reveal two structural phase transitions.^57^ These transitions occur around 12.9 and 17.5 GPa, respectively. The transitions involve a collapse of the unit-cell volume and an increase in the coordination number of the Sn and W atoms. This suggests a densification of the structure and a change in the bonding environment of the Sn and W atoms. In particular, the pressure-driven transitions suppress the lone-pair stereochemical activity of Sn^2+^. One of the HP phases of SnWO_4_ is isostructural to the PbWO_4_–III structure^57^ found under HP in other AWO_4_ tungstates. Under compression, metastable β-SnWO4 decomposes into Sn, SnO_2_, and WO_3_.^58^ This decomposition occurs at a pressure of 14 GPa and is irreversible. The decomposition is likely due to the instability of the β-SnWO_4_ structure under pressure, potentially caused by the need for a change in Sn coordination from octahedral to tetrahedral during the transition to α-SnWO_4_, which is the most stable structure.

AlWO_4_ and CrWO_4_ have not been previously examined under compression. In this article, we will introduce the initial findings from DFT calculations conducted for these compounds. Additionally, we will provide the results of calculations performed for BeWO_4_, which is another compound that has not been studied so far.

## 4 Changes Induced by Pressure in the Crystal Structure

### 4.1 Linear Compressibility of Axes

In this section we will discuss the changes induced by pressure in the scheelite and wolframite structures. We will discuss the linear compressibility of crystallographic axis and the volumetric compressibility. In Figure 3, we present the pressure dependence of the unit-cell parameters of one scheelite (CaWO_4_) and one wolframite (FeWO_4_). We choose these two compounds as representatives of the two families of compounds.

**Figure 3:**
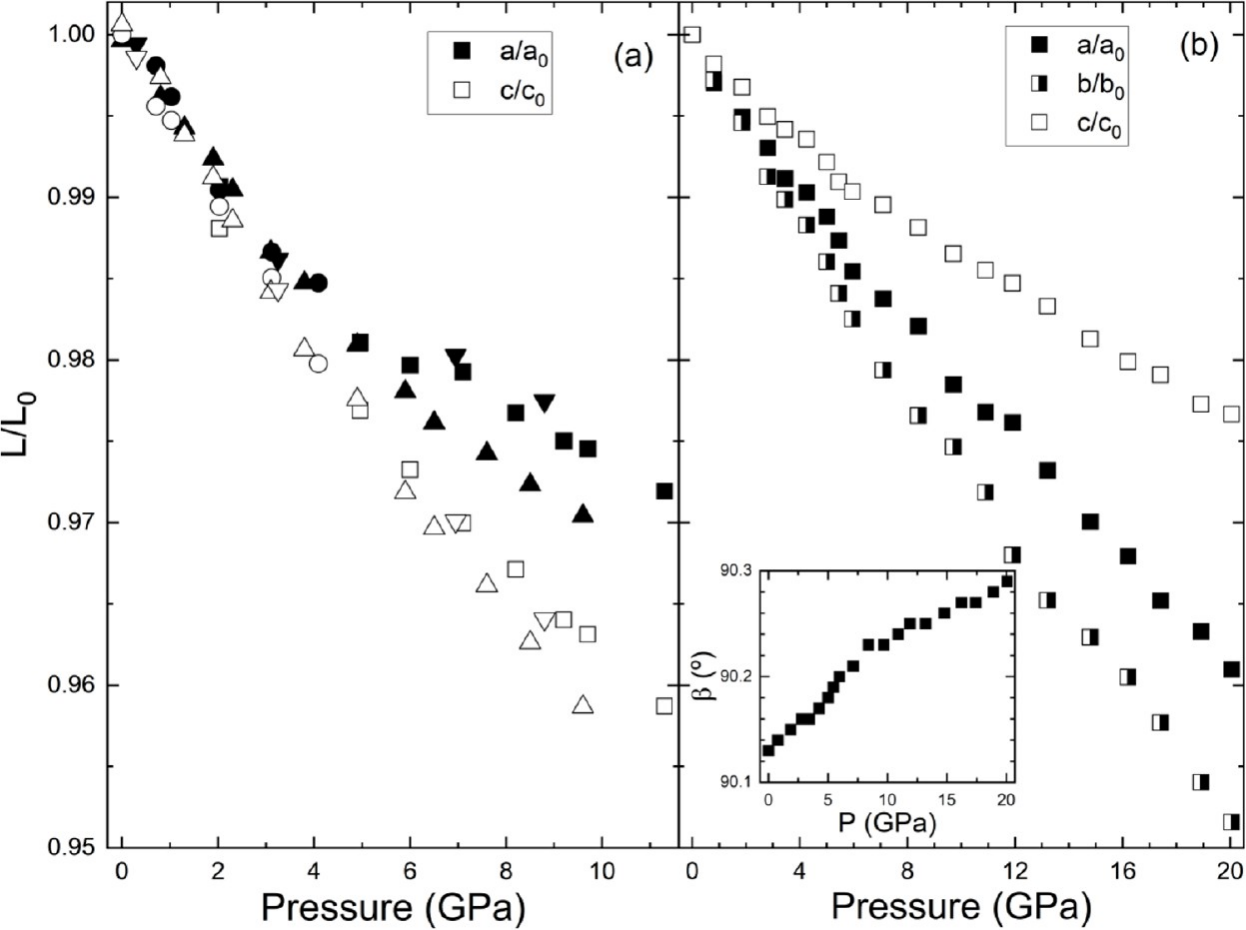
Relative change of unit-cell parameters (*L*/*L*_0_) with pressure. In (a), we show results for CaWO_4_. Circles are from ref 16, up triangles from ref 27, down triangles from ref 34 and squares from ref 28. Copyright 2005 American Physical Society. In (b), we show results for FeWO_4_ taken from ref 8. The inset shows the pressure behavior of the β angle of wolframite FeWO_4_. Copyright 2024 American Chemical Society.

Figure 3a presents the results measured for CaWO_4_ in four independent experiments.^16,27,28,34^ Despite the data scattering, caused by the differences in experimental conditions, the four experiments show a similar qualitative behavior. The figure shows that the compressibility of the *c*-axis in the scheelite structure is greater than the compressibility of the *a*-axis. This behavior has been also found in all the other scheelite tungstates and molybdates studied under pressure until now.^1,16,27,28,34,44,59–62^ The linear compressibility of each axis determined from XRD experiments^16^ is κ_a_ = 4.2 × 10^–3^ GPa^–1^ and κ_c_ = 5.2 × 10^–3^ GPa^–1^. This observation is consistent with the elastic constant measured using ultrasound techniques,^63^ which found that C_11_ > C_33_. The linear compressibility values obtained from the elastic constants are κ_a_ = 3.8 × 10^–3^ GPa^–1^ and κ_c_ = 4.8 × 10^–3^ GPa^–1^. The values measured for the elastic constants related to shear deformations^63^ show that CaWO_4_ is easily deformable under shear stress, which explains why nonhydrostatic conditions might affect the HP behavior as discussed in the previous section. The anisotropic compressibility of scheelite-type oxides has been explained because WO_4_ and MoO_4_ tetrahedra are more rigid units than AO_8_ dodecahedra.^1^ The AO_8_ units are connected by shared edges to other AO_8_ units forming zigzag chains running along the *c*-axis. These chains are separated by WO_4_ tetrahedra. Consequently, along the *a*-axis there is a sequence of AO_8_–WO_4_-AO_8_–WO_4_··· units. Then, due to the larger compressibility of AO_8_ dodecahedra compared to WO_4_ tetrahedra, the *c*-axis is the most compressible axis of scheelites.

Figure 3b shows the pressure dependence of the lattice parameters of FeWO_4_. The behavior of FeWO_4_ is representative of that of all wolframite-type tungstates. These compounds exhibit an anisotropic behavior under pressure. This means that their compressibility varies along different crystallographic axes. Specifically, the *b*-axis of wolframite contracts more significantly than the *a* and *c* axes under pressure; see Figure 3b. This anisotropic compression is due to the arrangement of WO_6_ and AO_6_ octahedra within the wolframite structure.^18,19^ The compressibility is also influenced by the different compressibilities of the individual octahedra and their arrangement; with the AO_6_ octahedra being more compressible than the WO_6_ octahedra.^15^ Another feature of wolframites is that the monoclinic β angle increases under compression and makes the crystal structure more distorted as pressure increases.

Since the crystal structure of wolframites is monoclinic, a more detailed understanding of their axial compressibility is derived from the examination of its compressibility tensor. In monoclinic structures, this tensor is symmetric and has only four nonzero components.^64^ The eigenvectors of the compressibility tensor are the main compressibility axes of the structure, and the corresponding eigenvalue is their compressibility. In the case of FeWO_4_, the main compressibility axes are (0,1,0), (10,0,1), (1,0, $\overset{-}{10}$ ). The values of the linear compressibility of these axes are 2.33(5) × 10^–3^, 1.92(2) × 10^–3^, and 1.09(1) × 10^–3^ GPa^–1^, respectively. The most compressible axis aligns with the unique crystallographic *b*-axis, while the other two define a plane that is perpendicular to it. This is a typical feature of all wolframites. Conversely, the direction exhibiting the least compressibility is contained within the *ac* plane, forming an angle of approximately 5° with the *c*-axis, measured from the *c*-axis toward the *a*-axis.

The behavior of pseudowolframite CuWO_4_ is also anisotropic. The pressure dependence of unit-cell parameters is shown in Figure 4 as obtained from two different powder XRD experiments, which have similar results.^60^ The compressibility of the axes decreases following the sequence *a*> *b*> *c*. Interestingly, those cell parameters that change most under compression are also those with larger thermal expansivity.^19^ Such correlation is also found in scheelites and wolframites.^1^ On the other hand, the α and β angle decrease under compression while the γ angle increases. This way the three angles approach 90° as pressure increases. The changes induced by pressure in the low-pressure triclinic structure of CuWO_4_ gradually make the structure more symmetric. This affects not only the global symmetry of the crystal but the local symmetry with the Jahn–Teller distortion of the CuO_6_ octahedron being reduced from 0.48(1) Å at ambient pressure to 0.37(1) Å at 7 GPa. Hence, compression induces a 23% reduction of the Jahn–Teller distortion of CuO_6_ before the onset of the phase-transition to the high-pressure monoclinic phase takes place. Interestingly, the onset of phase transition is related to the fact that reduction of the Jahn–Teller distortion reaches its limit value.

**Figure 4:**
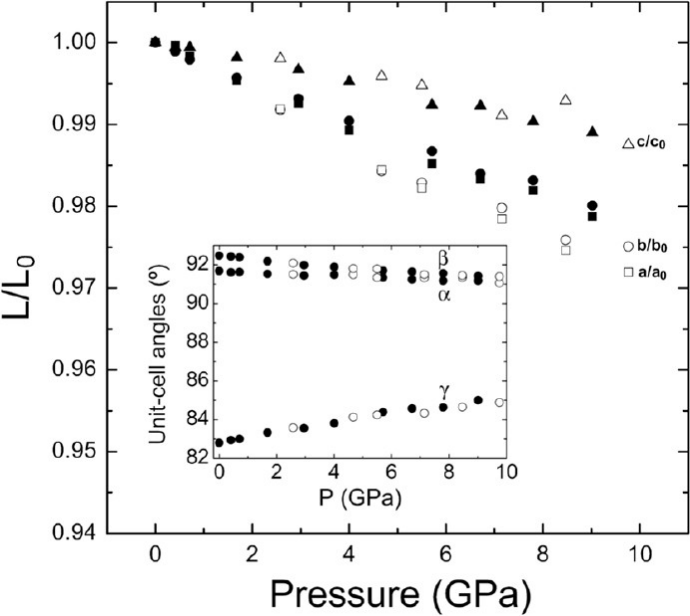
Relative change of unit-cell parameters (*L*/*L*_0_) with pressure in CuWO_4_. Solid symbols are from experiments performed using silicone oil as pressure medium, and empty symbols are from experiments performed using argon as pressure medium.^60^ The inset shows the pressure behavior of the angles. The figure is adapted with permission from ref 60. Copyright 2010 American Physical Society.

HgWO_4_ has an anisotropic behavior under compression.^56^ In this compound, interestingly, the *b*- and *c*-axis are reduced when pressure increases. The *b*-axis is the most compressible axis. In contrast, the *a*-axis expands under pressure. On the other hand, the β angle changes from 113° to 116° from ambient pressure to 16 GPa.^56^ This anisotropic behavior is attributed to the greater compressibility of the HgO_6_ octahedra compared to that of the WO_6_ octahedra, as well as to an octahedral tilting induced by pressure.^56^ A clearer visualization of the changes induced by pressure in HgWO_4_ is achieved when the crystal structure is described using the space group *I*2/*a* instead of *C*2/*c*. The transformation is *a̅* ^’^ = *c̅*, *b̅* ^’^ = *b̅*, and *c̅* ^’^ = −(*a̅* + *c̅*), where the primed unit-cell vectors are for space group *I*2/*a* and the unprimed for space group *C*2/*c*. After this transformation the unit-cell parameters change from *a* = 11.335 Å, *b* = 6.021 Å, *c* = 5.148 Å, and β = 113.11° to *a’* = 5.148 Å, *b’* = 6.021 Å, *c’* = 10.476 Å, and β*’* = 93.76°. Using space group *I*2/*a* to describe HgWO_4_, it is more evident that as pressure increases, the crystal structure of HgWO_4_ becomes gradually more symmetric. At 15 GPa, the β*’* angle becomes very close to 90° and *c’* ≅ 2*a’*. In fact, the structure at 15 GPa can be described as pseudo-orthorhombic. The symmetrization of the crystal structure of HgWO_4_ results in the gradual merging of XRD peaks.^56^ The differences of the behavior of HgWO_4_ compared to the wolframites can be ascribed to the peculiar crystal chemistry of Hg.^65^ Unlike transition metals, mercury exhibits distinctive bonding characteristics that influence the structure and properties of its compounds.^23^ Specifically, the incorporation of Hg into the structure of AWO_4_ compounds alters the electron distribution and local coordination environment, leading to a distinctive HP behavior.

In α-SnWO_4_, the compressibility is also anisotropic.^57^ According to XRD experiments and DFT calculations, the *a*- and *c*-axis are 50% more compressible than the *b*-axis. This is related to the orientation of the lone electron pairs of the Sn atoms. The smaller compressibility of the *b*-axis is related to the fact that changes induced by pressure in the crystal structure tend to minimize repulsions between electron pairs and favor the interaction of the lone electro pairs with the surrounding O atoms. The linear compressibility of the three crystallographic axes α-SnWO_4_ are κ_a_ = 3.2 × 10^–3^ GPa^–1^, κ_b_ = 2.1 × 10^–3^ GPa^–1^, and κ_c_ = 3.4 × 10^–3^ GPa^–1^.

### 4.2 Bulk Modulus

We will discuss next the pressure dependence of the unit-cell volume of divalent tungstates and their pressure–volume equation of state (EoS) at room-temperature. High-pressure EoS are crucial for understanding the behavior of materials under extreme conditions. The pressure dependence of all studied scheelites and wolframites is summarized in Figure S3 in the SI. The figure also includes results for CuWO_4_, HgWO_4_, and SnWO_4_. To facilitate comparison, the volume (V) has been normalized by the volume at ambient pressure (*V*_0_). The dependence of the volume for each compound shown in the figure corresponds to the EoS given in Table S1 in the SI, which will be discussed in the next paragraph. Figure S3 (SI) shows that scheelites, HgWO_4_, and SnWO_4_ (Figure S3a in the SI) are more compressible than wolframites and pseudowolframite CuWO_4_ (Figure S3b in the SI). These compounds require a pressure of approximately 20 GPa to reach the same relative change in the volume as the other compounds at 10 GPa.

In all the compounds here discussed, the pressure dependence of the volume is well described using a third-order Birch–Murnaghan EoS,^66^ which was developed based on the theory of finite strain.^67^ This EoS has three parameters viz. the volume at zero pressure (*V*_0_), the bulk modulus at zero pressure (*B*_0_), and its pressure derivative *B*_0_’. The values of these parameters for each compound here discussed, using the volume normalized per formula unit are summarized in Table S1 in the Supporting Information. Volumes have been normalized to allow a direct comparison between compounds that have two and four formula unit per unit cell. The values included in the table were taken as the average of values found in the literature.^8,16,19,27,28,34,38,43,44,50,52–54,56,57,59,60,68^

In Table S1 (SI) the readers can see that wolframites and CuWO_4_ have a larger bulk modulus than the rest of AWO_4_ compounds. This is consistent with the smaller compressibility of the first group of compounds as shown in Figure S3 (SI). Table S1 (SI) suggests that there is an apparent inverse correlation between the normalized unit-cell volume and the bulk modulus. Such relationship has been proposed for alkali-halide and tetrahedral semiconductors.^69^ However, as we will show next, it does not hold for divalent tungstates. This can be seen in Figure 5a. This figure shows that there is no clear analytical relation between the bulk modulus and the unit-cell volume. It appears that there are two different behaviors, one for the compounds with smaller volumes, those going from NiWO_4_ to CdWO_4_ in Table S1 (SI), and another for the compounds with a larger volume, those from CaWO_4_ to BaWO_4_ in Table S1 (SI). It is noticeable the large reduction of the bulk modulus when comparing CdWO_4_ to CaWO_4_. A change in the volume from 74 to 78 Å^3^ (5.4%), causes a decrease of the bulk modulus from 123 to 73 GPa (41%). Therefore, it is thus evident that the bulk modulus of all divalent AWO_4_ tungstates cannot be simply correlated to the unit-cell volume.

**Figure 5:**
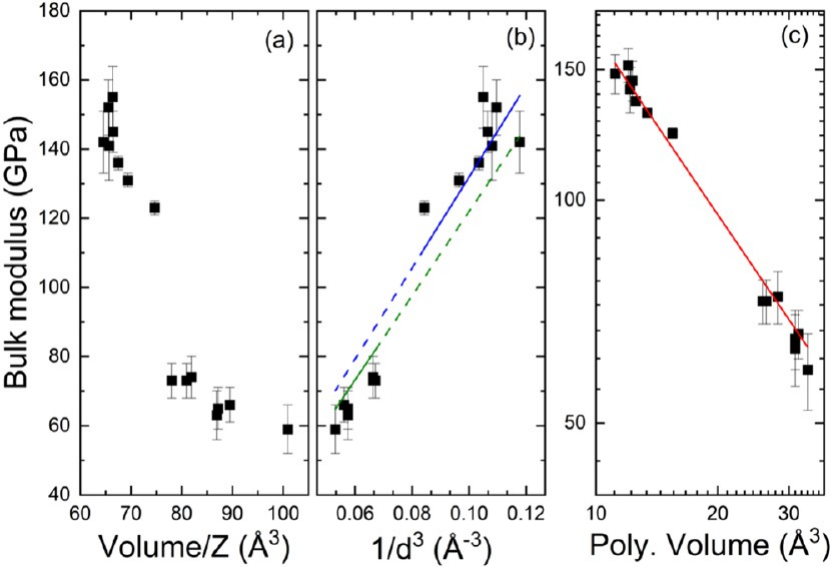
(a) Bulk modulus versus volume per formula unit (*Z*). (b) Bulk modulus versus 1/*d* ^3^, where *d* is the average bond distance of the A–O bonds within the coordination polyhedron of the A cation. The green and blue solid (and dashed) lines are the relationships discussed in the text. (c) Bulk modulus versus the volume (log–log scale) of the coordination polyhedron of the A cation. The red line is the relationship described in the text.

In the case of ternary oxides related to the tungstates, it has been proposed that the compression experienced by them primarily results from the shortening of the larger cation-oxygen bonds, rather than by alterations in the shorter cation-oxygen bonds.^70,71^ For instance, in ZrSiO_4_ large Zr–O are highly compressible and short Si–O bonds are incompressible.^72^ Thus, the shortening of Zr–O bonds, as a first approximation, determines the compressibility of ZrSiO_4_. Analogously, in AWO_4_ compounds, the behavior of A–O is expected to be determinant in the compressibility of these tungstates. Hazen and Finger tried to establish a connection between the bulk and bond compressibility in AMO_4_ compounds.^70^ They suggested that the bulk modulus of these compounds is directly correlated to the compressibility of the A–O bonds found within the A-cation coordination polyhedron. Specifically, they proposed that *B*_0_ (in GPa) equals 750 *Z_i_*/*d* ^3^, where *Z_i_* represents the cationic formal charge (in our compounds *Z_i_* = 2) and *d* denotes the mean value of the A–O bond distance (in Å). More recently, Errandonea and Manjón^1^ extended the number of compounds included in the analysis and found that in AMO_4_ compounds, including scheelite-type tungstates, the relation is *B*_0_ = 610 *Z_i_*/*d* ^3^. On the other hand, Errandonea and Ruiz-Fuertes^18^ proposed that in wolframites the relation is 660 *Z_i_*/*d* ^3^. However, it looks unintuitive that two groups of compounds of the same family would follow a different law to explain their compressibility.

In Figure 5b we represent the bulk modulus of all AWO_4_ compounds summarized in Table S1 (SI) as a function of 1/*d* ^3^. The figure shows that in fact, wolframite-type compounds follow a different behavior than the rest of the compounds. In the figure, the relationships proposed before for scheelites^1^ and wolframite^18^ are shown by green and blue lines, respectively. The extrapolation of either of these lines does not fit the data of the other part of the tunsgtate family as shown by the dashed lines shown in Figure 5b. A possible explanation to this could be the fact that, to simplify the model, Hazen and Finger^70^ and Errandonea et al.^1,18^ considered average bond distances within the coordination polyhedra of A cations and not the polyhedral volume. Scheelites and wolframites have different coordination polyhedral units for the A cation, AO_8_ bisdisphenoids in scheelites and AO_6_ octahedra in wolframites. Trying to better understand the bulk modulus of AWO_4_ compounds, we have followed the approach proposed by Anderson and Nafe^73^ and represented in Figure 5c the bulk modulus versus the inverse of the volume of the coordination polyhedra of the A cation. In the case of SnWO_4_ we used the volume of the distorted octahedron formed when considering the coordination of Sn as 4 + 2, which is the coordination formed under HP. In Figure 5c we used a log–log scale to linearize the relationship. The polyhedral volumes were obtained from the crystal structures using VESTA.^74^ The figure shows that in this representation all compounds can be described by a single relationship. We found that the best fit is given by 1064(108) × *V* ^–0.80(3)^, where *V* is the polyhedral volume in Å^3^. The fit is shown with a red line in Figure 5c, the *R*-square of the fit is 0.98 and the reduced χ^2^ is 1.07. This result shows that the bulk modulus of AWO_4_ compounds is determined by the size of the AO_6_ or AO_8_ polyhedra independently if they are octahedra or dodecahedra. We have also found that the relationship here proposed for the bulk modulus also works well with rare-earth orthotungstates with formula RE_2_(WO_4_)_3_, where RE represents a rare-earth element. These compounds have a crystal structure which is a distorted supercell of scheelite. The relationship here proposed agrees within 10% with the bulk modulus measured by Sabalisck et al. for ten different RE_2_(WO_4_)_3_ compounds^75^ and might be considered a good approximation for the bulk modulus of tungstates not studied yet under compression.

## 5 Band Gap and Its Pressure Dependence

AWO_4_ tungstates are semiconductor materials with tunable band gaps, making them suitable for various applications in optoelectronics, photocatalysis, and energy storage. Tungstates with smaller band gaps: (e.g., SnWO_4_, FeWO_4_) can absorb a wider range of visible light, making them effective for photocatalytic degradation of pollutants and water splitting. ZnWO_4_ is employed in wastewater treatment and degradation of organic pollutants. CoWO_4_ is a potential candidate for oxygen evolution and hydrogen reduction reactions in water splitting. This material is also being explored for use in supercapacitors and as cathode materials in lithium-ion batteries. CdWO_4_ and PbWO_4_ are used in radiation detectors due to their scintillation properties. MnWO_4_ exhibits multiferroic properties, making it suitable to be used in sensors and magnetoelectric devices. In addition, AWO_4_ compounds exhibit favorable optical properties and are utilized in lasers, photonic applications, and as phosphors in laser-emitting diodes. ZnWO_4_ is known for its photoluminescence and scintillation properties, making it useful in radiation detectors and biomedical applications. CaWO_4_ and ZnWO_4_ are being investigated for use in hybrid organic–inorganic X-ray detectors. The list of applications does not finish here and includes also other functions like the use in photodynamic therapy for cancer treatment. For many of the applications, it is important to know the accurate value of the band gap energy (*E*_gap_). It is also important to know the effect of pressure on the band gap. This section of the article is devoted to this subject.

We will start by reviewing and discussing the band gap energy at ambient conditions. We will focus on scheelites and wolframites, which have been studied at ambient pressure and under high pressure. Other compounds, like HgWO_4_ and SnWO_4_, will be discussed in next section. In Table S2 (SI) we summarize the band gap energy reported in the literature for each compound.^8,39,50,59,76–81^ Given the variation in the reported values of *E*_gap_ for each compound, we have chosen to focus our discussion on the results derived from optical absorption measurements, which are widely regarded as the most accurate method.^82^ In contrast, other techniques, such as diffuse reflectance measurements coupled with Tauc plot analysis generally underestimate the band gap energy.^83^

Let first discuss scheelite-type tungstates. When comparing them, two facts can be noticed. CaWO_4_, SrWO_4_, and BaWO_4_ have wide band gaps with *E*_gap_ > 4.9 eV while EuWO_4_ and PbWO_4_ have *E*_gap_ = 4.01(1) and 3.1(1) eV, respectively. On the other hand, the three alkaline-earth tungstates are direct gap materials, while the other two compounds have an indirect band gap.^39,79^ As we will discuss below, the distinction is primarily related to the different contributions of orbitals from the divalent cation to the states that are in proximity to the valence band maximum and the conduction band minimum.

To compare alkaline-earth tungstates with the other scheelite-type tungstates, we will use CaWO_4_ and PbWO_4_. The band structures are represented in Figure 6 and the electronic density of states in Figure 7. The results were obtained from our PBEsol calculations, which slightly underestimate the band gap energy of both compounds, but allow for a systematic comparison between them. Both figures show the qualitative differences between the band structure and electronic density of states for both compounds. They will be discussed in the following paragraphs. These differences will have an impact on the high-pressure behavior of the band gap as we will discuss in this section.

**Figure 6:**
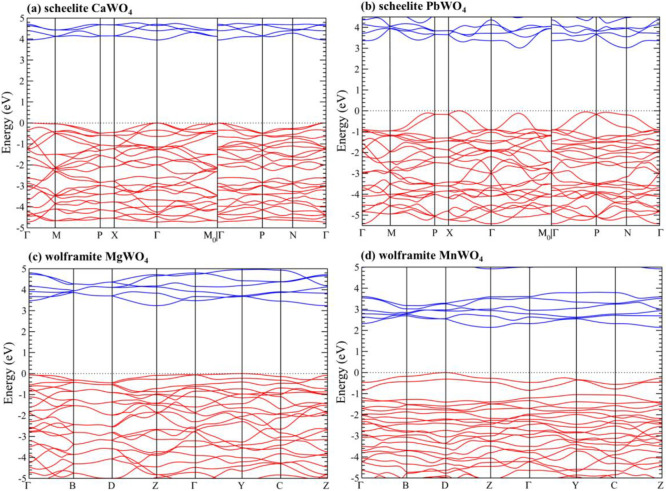
Band structures calculated in this work at ambient pressure using PBEsol for (a) CaWO_4_, (b) PbWO_4_, (c) MgWO_4_, and (d) MnWO_4_.

**Figure 7:**
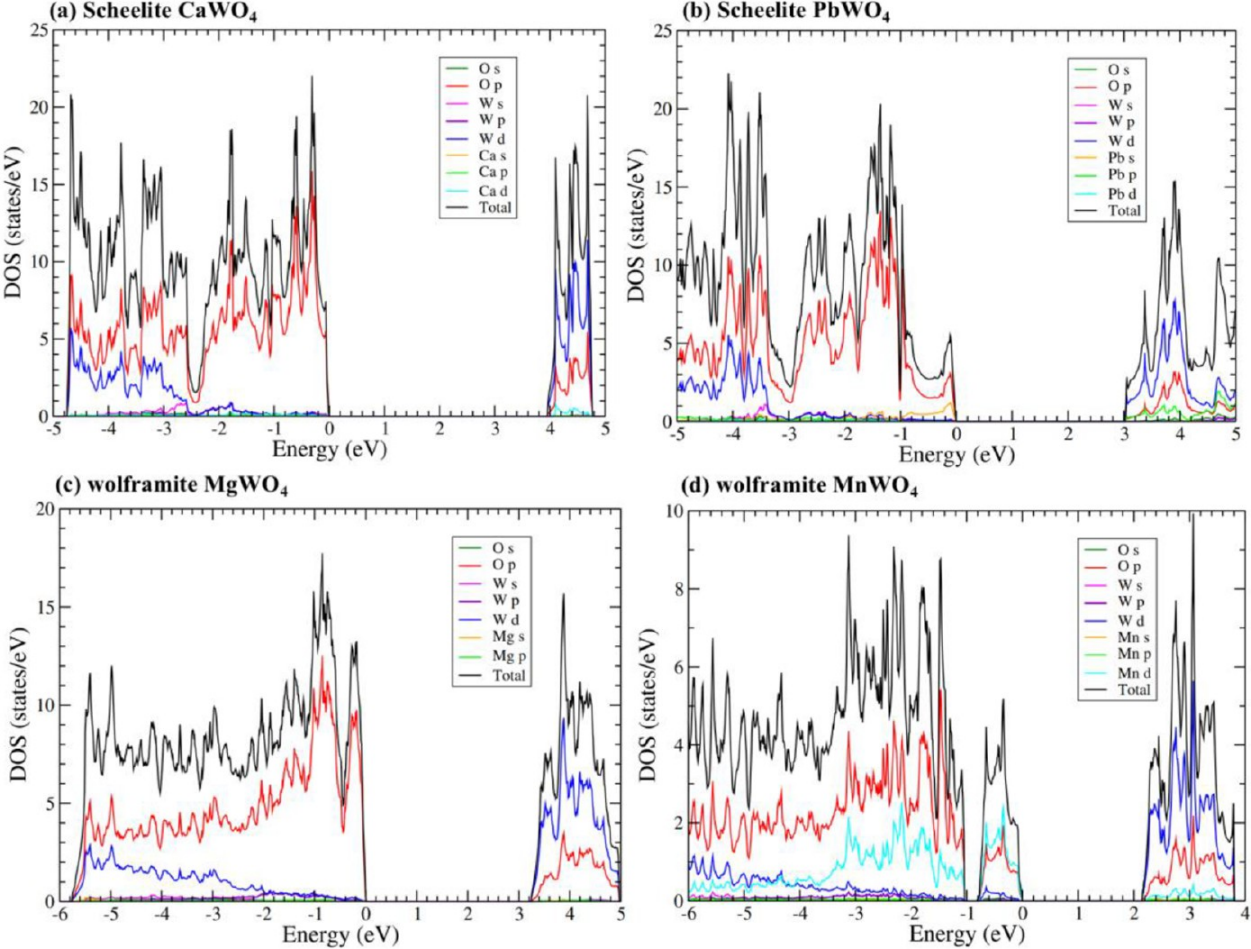
Electronic density of states calculated in this work at ambient pressure using PBEsol for (a) CaWO_4_, (b) PbWO_4_, (c) MgWO_4_, and (d) MnWO_4_.

The calculated electronic density of states of CaWO_4_ (see Figure 7a) shows that in alkaline-earth tungstates, the upper section of the valence band and the lower section of the conduction band are largely determined by the WO_4_ tetrahedra.^84^ The W 5*d* states interact with the 2*p* states of the surrounding O ions, leading to the formation of bonding and antibonding orbitals that contribute to the valence and conduction bands, respectively. As shown in Figure 6a, in CaWO_4_, the maximum of the valence band (VBM) and the minimum of the conduction band (CBM) are at the center of the Brillouin zone (Γ point). This is because the symmetry of the crystal structure constrains the possible energy levels and wave functions of the electrons. The fact that the band gap is direct is important for optoelectronic applications.^85^ Another important characteristic of the band structure is that the dispersion of the valence bands is relatively small, with comparable dispersions along different directions. The top of the valence band is mainly contributed by O 2*p* states. On the other hand, the lower part of the conduction band, which is composed primarily of *e*_g_ states associated with the W 5*d* states, with some contribution of O 2*p* states, is separated by approximately 0.5 eV from the upper part of the conduction band formed from the hybridized *t*_2_ states and the 3*d* states of Ca, 4*d* states of Sr, and 5*d* states of Ba. The fact that only 5*d* states from W and 2*p* states from oxygen contribute to the bottom of the conduction band and the top of the valence band explains why alkaline-earth tungstates have a band gap with an energy larger than 4.9 eV.

The band structure of PbWO_4_, shown in Figure 6b, exhibits notable differences compared to the alkaline-earth tungstates.^79,85^ In PbWO_4_, which is an indirect semiconductor, the extrema of the bands are situated away from the Γ point. In PbWO_4_ the valence band reaches its absolute maximum at a point between X and Γ (named Δ), however, there are additional local maxima, also away from the Γ point, which are nearly degenerated in energy with the absolute maximum. On the other hand, the absolute minimum of the conduction band is found in a point between Γ and *M*_0_ (named Σ). The different topology of the band structure of PbWO_4_ is related to the contribution of Pb 5*d* and 6s states to the top of the valence band and the bottom of the conduction band (see Figure 7b), which is a distinctive characteristic of PbWO_4_. This fact causes PbWO_4_ to display a smaller band gap compared to CaWO_4_, SrWO_4_, and BaWO_4_. Basically, the O 2*p* states and W 5*d* states hybridize with the 6*s* and 5*d* states of Pb, causing a reduction of the band gap. From a symmetry perspective, it is anticipated that the Pb 6*s* and O 2*p* states will not mix at the Γ point but will exhibit strong mixing in directions with reduced symmetry. Because of it the band gap in PbWO_4_ is indirect. In the case of EuWO_4_, the gap is indirect and smaller than in alkaline-earth tungstates due to the influence of Eu 4*f* electrons.^39,81^ In this compound, the VBM is at the X point of the Brillouin zone, and it is dominated by 4*f* states from Eu. On the other hand, the CBM is at Γ by mostly contributed by 5*d* states from W. In EuWO_4_, the 2*p* states from O are situated around −2 eV below the VBM.

The differences in the orbital composition of the band structures of EuWO_4_ and PbWO_4_ with alkaline-earth tungstates explain the different behavior of the band gap under compression. The band gap of PbWO_4_ closes with pressure due to the unique electronic configuration of lead, where its 6*s* states contribute to antibonding orbitals at the top of the valence band, causing the band gap to shrink as the lattice compresses. An analogous behavior occurs in EuWO_4_ due to the contribution of Eu 4*f* states to the top of the valence band. In contrast, the band gap of CaWO_4_, SrWO_4_, and BaWO_4_ than band gap is slightly affected by pressure because its valence band top is more stable because the states near the Fermi level are dominated by W 5*d* and O 2*p* states.^79^

We will now discuss wolframite compounds. The band structures of MgWO_4_, CdWO_4_, and ZnWO_4_, three compounds where the divalent cation has a filled *d* electron shell, exhibit significant similarity at both ambient pressure and under compression.^77^ Consequently, we will focus on the band structure of MgWO_4_. Its band structure and electronic density of states are presented in Figures 6c and 7c, respectively. Concerning the atomic contributions of the VBM and the CVM, they are comparable to those observed in CaWO_4_, where the dominant states are attributed to O 2*p* and W 5*d*. The contribution from Mg states is minimal. The conduction band has mainly a *d* character (specifically W 5*d*). Consequently, the conduction band is not highly dispersive, nor is its energy minimum located at the center zone. Furthermore, since wolframites crystallize in a monoclinic *P*2/*c* space group, which is centrosymmetric, *p*-*d* mixing does not occur at the Γ point; however, it does occur at less symmetrical points, resulting in upward (downward) dispersion in the valence (conduction) band when transitioning from the Γ point. This necessarily indicates that if a wolframite possesses a direct band gap, it must occur away from the zone center, which is indeed the case for MgWO_4_, ZnWO_4_, and CdWO_4_, which exhibit direct band gaps at the Z point (see Figure 6c).

In compounds like CoWO_4_, NiWO_4_, CuWO_4_, FeWO_4_, and MnWO_4_, where the divalent cation has a partially filled *d* electron shell, the band structure is different. This is because there is no *p*-*d* mixing in Γ, but there is *p*-*d* mixing in lower symmetry points away from Γ.^77^ The *p*-*d* repulsion leads to an upward dispersion of the valence band, resulting in the maximum occurring at the edge of the Brillouin zone. Specifically, in the case of MnWO_4_, from the band structure shown in Figure 6d, it is evident that the indirect band gap arises from the top of the valence band at point D and the bottom of the conduction band at point Z. This phenomenon is attributed to the 3*d* states of Mn, which are hybridized with the O 2*p* states.^77^ A consequence of the important contribution of Mn 3*d* orbitals to the states near the Fermi level (see Figure 7d) is the fact that magnetic wolframites with transition metals as divalent cation, as those discussed in this paragraph, have a band gap energy smaller than MgWO_4_, ZnWO_4_, and CdWO_4_, as shown in Table S2 (SI).

Under compression, a contrasting behavior is noted between the two groups of wolframites. In compounds like MgWO_4_, the energy level of the conduction band bottom increases with pressure, while for compounds like MnWO_4_, it decreases. For both kinds of materials, the energy level of the top of the valence band remains unchanged. This results in an increase in the band gap for MgWO_4_, ZnWO_4_, and CdWO_4_ and a decrease for the rest of wolframites. The electronic density of states of MgWO_4_ shows that the lower region of the conduction band in wolframite orthotungstates is primarily influenced by contributions from W 5*d* states. Conversely, the upper section of the valence band is predominantly made up of O 2*p* states. In compounds where the valence shell of the divalent cation consists solely of s or fully filled *d* states, their contribution to the valence and conduction bands is minimal. However, when the divalent cations possess an open *d* shell, a more significant contribution from the divalent metal to both the valence and conduction bands is noted. As a result, the reduction in the band gap of compounds like MnWO_4_ observed under compression can be qualitatively accounted for by considering the increase in pressure of the crystal field affecting the W 5*d* and O2*p* states, along with the resulting enhancement of their hybridization with the states of the divalent metal.^77^

## 6 Previously Unstudied AWO_4_ Compounds

### 6.1 BeWO_4_

Among AWO_4_ materials, the most elusive piece is beryllium tungstate, BeWO_4_. This is partly because the formation of BeWO_4_ is less favored than the formation of other alkaline earth orthotungstates by the electronegativity order of the oxides: BaO < SrO < CaO < MgO < BeO.^86^ However, it has been reported that above 2100 K, beryllium oxide reacts with tungsten oxide, forming BeWO_4_ in energetic environments.^87^ It has also been found that after vaporization, BeO reacts with tungsten, forming different beryllium tungstate oxide spices.^88^ The formation of BeWO_4_ and the knowledge of its physical properties have very important implications for environments with a high heat load, for instance, in turbines or reactors. Beryllium and tungsten surfaces play an important role in nuclear fusion devices like the Joint European Torus (JET) and the Thermonuclear Experimental Reactor (ITER).^88,89^ It has been proposed that the interaction of energetic oxygen ions with the beryllium–tungsten alloy Be_2_W favors the formation of BeWO_4_.^90^ The current lack of knowledge of the physical properties of this compound could result in catastrophic events. For instance, a melting temperature much lower than that of W, 3600 K,^91^ or a low value of the bulk and/or shear modulus would eventually lead to the failure of components. In the case of fusion reactors, ignorance of physical properties could be a severe safety issue since the formation of volatile tungsten oxides would lead to potential escapes of radioactivity.^90^ Unfortunately, up to now BeWO_4_ has not been produced in sufficient amount for the crystal structure to be determined. Not only is the crystal structure unknown, but also the rest of the physical properties, including the elastic moduli and phonon frequencies.

To circumvent experimental limitations that have prevented the characterization of BeWO_4_ we have studied it by means of density-functional theory simulations. Through structural optimization calculations, we have explored different candidate structures aiming to determine the most likely structure of BeWO_4_. We used the Vienna Ab initio Simulation Package (VASP).^92^ The exchange–correlation energy has been described within the framework of the generalized-gradient approximation (GGA)^93^ Using the Perdew–Burke–Ernzerhof functional (PBEsol).^94^ A cutoff energy of 560 eV was taken in the plane waves expansion. The conventional Monkhorst–Pack scheme^95^ was used for the k-space summations within the Brillouin zone with a 6 × 6 × 6 grid. Dynamical properties were studied with the Phonopy package^96^ using a 4 × 4 × 4 supercell. The crystal structures considered in calculations were selected using crystal chemistry arguments. They include all those observed in divalent metal tungstates at ambient and high-pressure, zircon, AgClO_4_, and CrVO_4_.

According to our calculations, the crystal structure with the lowest enthalpy at ambient conditions is a triclinic structure (space group *P*1̅). The calculated unit-cell parameters of this structure are *a* = 4.999 Å, *b* = 5.157 Å, *c* = 5.658 Å, α = 66.19°, β = 88.03°, and γ = 81.67°. The calculated atomic positions are given in Table S3 (SI). The structure resembles that of CuWO_4_. However, BeWO_4_ has a much smaller α angle. In addition, BeWO_4_ forms a layered structure formed by WO_6_–BeO_4_–BeO_4_–WO_6_ chains, as shown in Figure 8. Notice that this is the only AWO_4_ compound where the divalent cation is in a 4-fold coordination forming BeO_4_ distorted tetrahedral units, with an average bond distance of 1.647 Å. Such coordination is typical for Be atoms in beryllium oxides and it is related to the small ionic radii of Be.^97^

**Figure 8:**
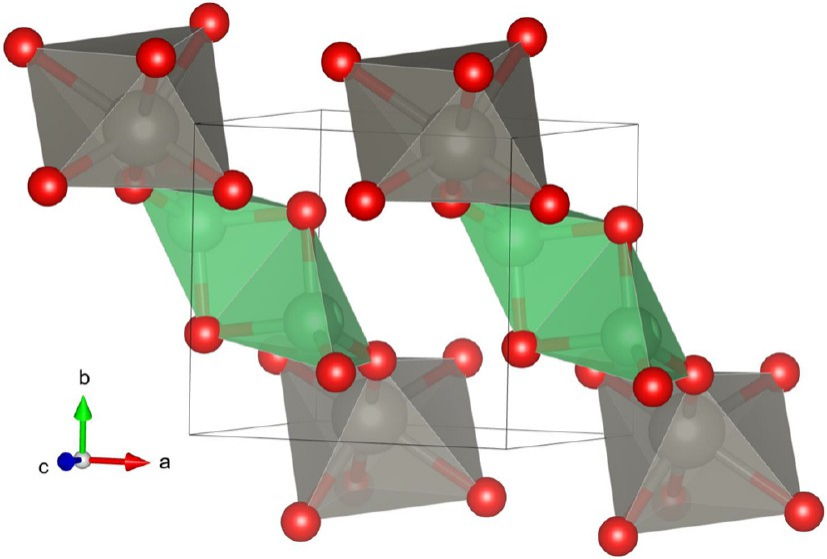
Crystal structure of BeWO_4_. The coordination octahedra (tetrahedra) of W (Be) are shown in gray (green). Oxygen atoms are represented in red. Black lines represent the unit cell.

An important result of our calculations is that, as shown in Figure 9, we found that the most stable structure of BeWO_4_ is energetically less favored than the coexistence of BeO + WO_3_. This result indicates that BeWO_4_ will likely decompose into BeO + WO_3_ at ambient conditions. Therefore, the detection of BeWO_4_, under ambient conditions, should be possible only as a metastable phase, which was obtained following its formation under extreme conditions and then recovered because of large energy barriers preventing the decomposition. A temperature of 2100 K was needed to form BeWO_4_ due to the large enthalpy of formation, 12.5 eV.^86^ The synthesis conditions resemble those of EuWO_4_ which is formed by the reaction of Eu_2_O_3_ and W at 2300 K.^38^ The recovery of BeWO_4_ as a metastable phase of BeWO_4_ is not an unusual phenomenon. Metastable phases formed under extreme conditions have been recovered in other oxides.^97,98^

**Figure 9:**
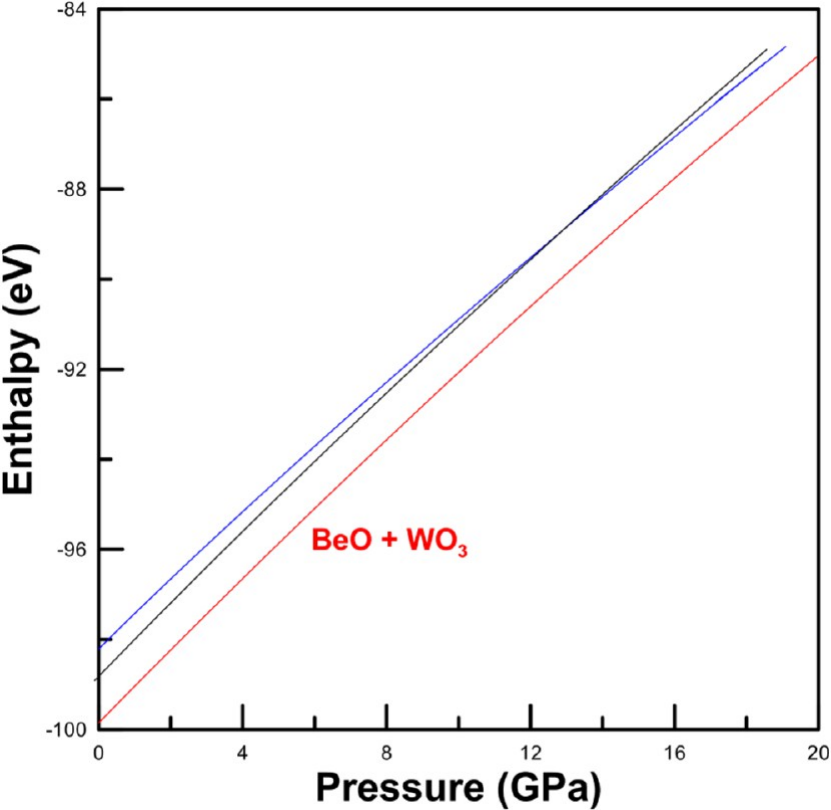
Calculated enthalpy per formula unit of BeWO_4_ and BeO + WO_3_ as a function of pressure. The black line is the enthalpy of the triclinic phase. The blue line is the enthalpy of a monoclinic wolframite-type phase. The red line is the enthalpy of the BeO + WO_3_ decomposition.

The elastic constants and phonon frequency dispersion spectrum have been calculated to ascertain the mechanical and dynamic stability of metastable BeWO_4_. The phonon dispersion for triclinic BeWO_4_ is illustrated in Figure S4 in the SI, which shows that all modes are real (positive). This finding supports the dynamical stability of the otherwise metastable BeWO_4_. From these calculations, we have also obtained the Raman-active and infrared-active phonons. The triclinic structure of BeWO_4_ has 18 Raman modes with *A*_g_ symmetry and 15 IR modes with *A*_u_ symmetry. The distribution of frequencies in the phonon spectrum is very similar to that of CuWO_4_ and wolframites,^99^ with the highest frequency modes being associated with internal vibrations of the WO_6_ octahedron. In particular, the modes at the highest frequencies are due to symmetric and asymmetric stretching vibrations. The calculated frequencies are summarized in Table S4 (SI).

Through the calculated elastic constants, we found that all eigenvalues of the elastic tensor are positive, indicating elastic stability for triclinic BeWO_4_. From the elastic constants, using the Hill approximation,^100^ we determined the bulk modulus, *B* = 80.9 GPa, shear modulus, *G* = 52.1 GPa, Young modulus, *E* = 128.7 GPa, and Poisson ratio, ν = 0.235. The *B*/*G* ratio is equal to 1.554. This and the value of ν suggest that BeWO_4_ is a brittle material.^101^ Regarding the pressure dependence of the volume, from our total-energy calculations, we found that it can be described with a third-order Birch–Murnaghan EOS with parameters *V*_0_ = 132.2 Å^3^, *B*_0_ = 72.3 GPa, and *B*_0_’ = 4.5. The two methods constrain the bulk modulus between 72.3 and 80.9 GPa. This means that BeWO_4_ is less compressible than scheelites but more compressible than wolframites. The fact that BeWO_4_ is the most compressible wolframite tungstate is due to the unique layered characteristics of its crystal structure (see Figure 8).

### 6.2 AlWO_4_

AlWO_4_ is an aluminum tungstate with an unusual oxidation state, +5, for W. The most common aluminum tungstate is Al_2_(WO_4_)_3_. AlWO_4_ is synthesized by a solid-state reaction from the mixture of Al_2_O_3_, WO_3_ and WO_2_ at temperatures between 800 and 1000 °C.^24^ The crystal structure of this compound is monoclinic (space group *C*2/*m*)^24^ and can be considered as a rutile-like framework. As shown in Figure 1f, the structure is formed by AlO_6_ octahedra developing in the direction [010] alternately with analogous rows of WO_6_ octahedra. The structure of AlWO_4_ is closer to that of wolframites than to scheelites. AlWO_4_ was found to have diamagnetic and semiconductor behavior.^24^ The characteristics of this tungstate make it useful as a catalyst in various industrial processes, particularly in refining processes that use hydrogen and a catalyst to convert lower-quality crude oil fractions and bio-oils into cleaner, higher-value products by removing impurities like sulfur, nitrogen, and oxygen, requiring efficient catalysis.^102^ However, little is known about the properties of AlWO_4_ at ambient pressure, and nothing is known about its high-pressure behavior. Here, we will present results on the influence of pressure in the crystal structure of AlWO_4_, as well as a precise calculation of the band structure and electronic density of state. Results on the elastic constants and moduli will also be presented. For AlWO_4_ calculations were performed using the same methodology as for BeWO_4_. In AlWO_4_ we also calculated the band-structure. For these calculations, we used the HSE06 functional^103^ instead of PBEsol, because it gives a more accurate description of the band gap energy. The study of the band gap energy and its dependence on pressure is important as nothing is yet known about this parameter, which determines the electrical and optical properties of a material.

The crystal structure optimized at 0 GPa has the following unit-cell parameters *a* = 9.1349 Å, *b* = 5.7324 Å, *c* = 4.5714 Å, and β = 92.22°. They agree within 1% with the parameters determined from previous X-ray diffraction experiments, *a* = 9.069 Å, *b* = 5.705 Å, *c* = 4.541 Å, and β = 92.29°.^24^ This confirms that the method used in the calculations gives an accurate description of the crystal structure. We noticed that in the crystal structure, the arrangement of AlO_6_ and WO_6_ octahedra is analogous to that of the rutile lattice of TiO_2_.^104^ In fact, AlWO_4_ has a structure that can be described as having a close-packed arrangement of its oxide ions, specifically a distorted hexagonal close-packed lattice, with the Al and W cations occupying half of the octahedral holes within this anion framework.

The relation between AlWO_4_ and rutile can be seen in Figure 10 where we compare both structures. In fact, the crystal structure of AlWO_4_ can be described as a distortion of a supercell of TiTiO_4_, in which Ti atoms (with +4 valence) are substituted alternatively by Al atoms (with +3 valence) and W atoms (with +5 valence). The monoclinic distortion is a result of the charge imbalance between both cations.^105^ It is interesting to note that between the space group of rutile *P*4_2_/*mnm* and the space group of AlWO_4_ *C*2/*m*, there is a group-subgroup relationship; *P*4_2_/*mnm* ⊂ *Cmmm* ⊂ *C*2/*m*. In fact, if in AlWO_4_ β = 90° a rutile structure with the unit-cell doubled along the *c*-axis of rutile (the *b*-axis of AlWO_4_) and along the *a*-axis of rutile is obtained. Note also that in AlWO_4_ *c*/*a* = 1.9983.

**Figure 10:**
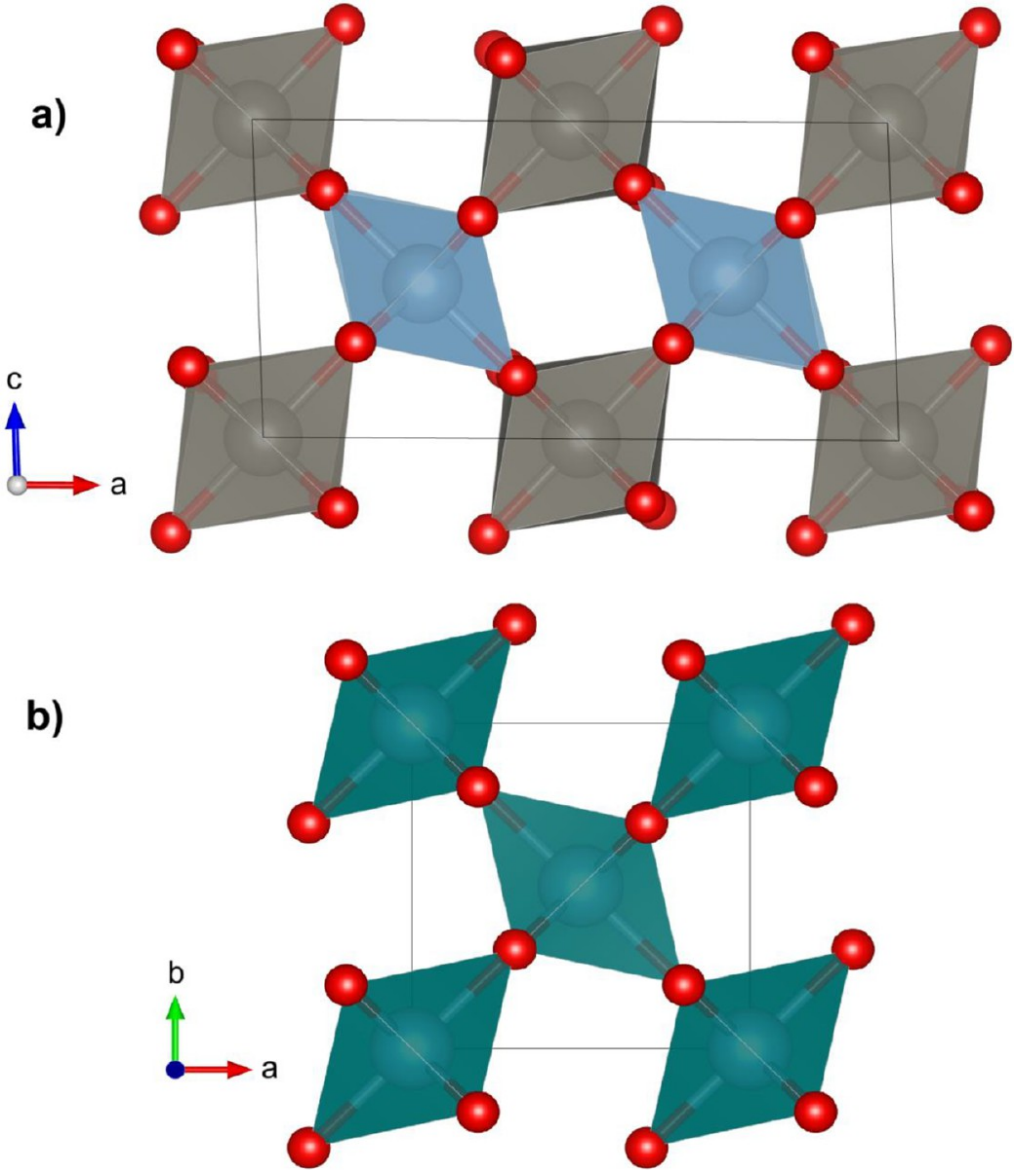
(a) Projection of the AlWO_4_ structure. (b) Projection of the TiO_2_ rutile structure. The projections were chosen to show the similarity between structures. AlO_6_, WO_6_, and TiO_6_ octahedra are shown in blue, gray, and green.

The elastic constants and the phonon dispersion spectrum have been computed to verify the mechanical and dynamic stabilities of AlWO_4_. The phonon dispersion for AlWO_4_ is depicted in Figure S5 in the SI. This figure demonstrates that all phonon branches display positive values. Such a result supports the dynamical stability of AlWO_4_. When calculating the elastic constants, we found that all eigenvalues of the elastic tensor are positive, which signifies elastic stability for AlWO_4_. We also found that the monoclinic structure of AlWO_4_ becomes mechanically unstable beyond 18 GPa. This result suggests that a phase transition might occur at 18 GPa. Utilizing the elastic constants and applying the Hill approximation,^100^ we calculated the bulk modulus, *B* = 254 GPa, shear modulus, *G* = 142 GPa, Young’s modulus, *E* = 359 GPa, and Poisson’s ratio, ν = 0.264. The *B*/*G* ratio is found to be 1.786. This ratio, along with the value of ν, indicates that AlWO_4_ is a brittle material.^101^

According to the calculations of the elastic constants, AlWO_4_ is highly uncompressible. The similarity between AlWO_4_ and rutile leads us also to think that AlWO_4_ is expected to possess a large bulk modulus, as most rutile-type oxides exhibit a bulk modulus exceeding 200 GPa.^106^ If we apply the empirical formula presented in Section 4.2, a bulk modulus of 220 GPa is obtained. From our DFT calculations, we determined the pressure dependence of the lattice parameters of AlWO_4_ up to 16 GPa, which is a pressure level below that at which mechanical instabilities arise and remains within the stability range of wolframite-type compounds. From these results, the pressure dependence of the volume is obtained. The results are shown in Figure 11.

**Figure 11:**
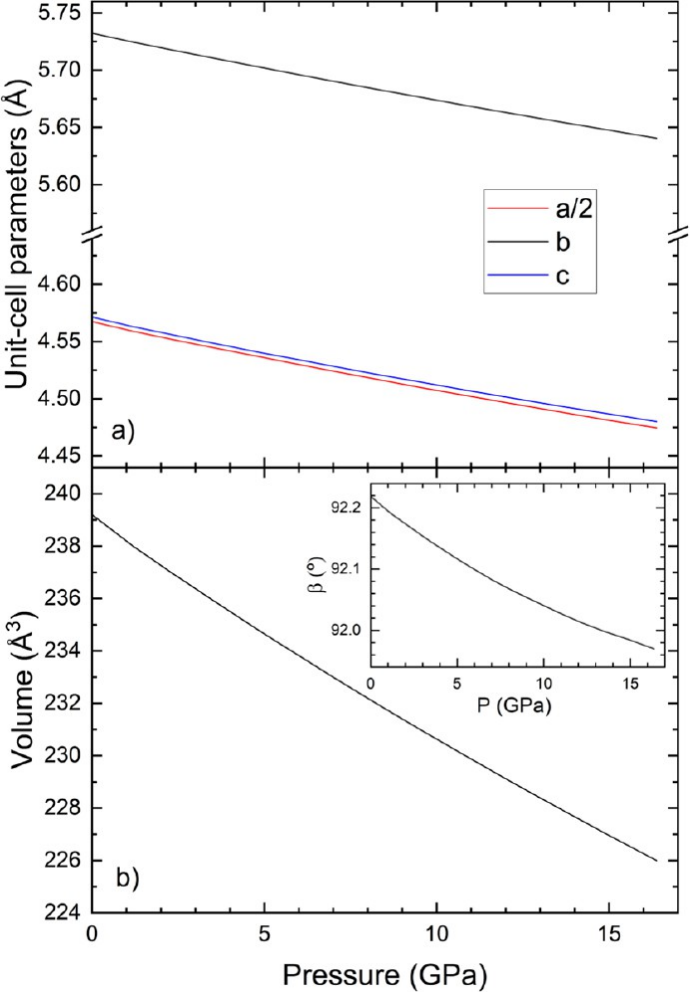
(a) Pressure dependence of the unit-cell parameters of AlWO_4_. We plot *a*/2 to facilitate comparison with the other axes. (b) Pressure dependence of the unit-cell volume. The inset shows the pressure dependence of the β angle.

From our results, we concluded that the compression of AlWO_4_ is nearly isotropic with the *b*-axis being slightly less compressible than the other two axes which have a very similar compressibility. We also found that the β angle is reduced under compression. Regarding the change of the volume with pressure, from 0 to 16 GPa, it decreases by 5.5%, which confirms that AlWO_4_ is as uncompressible as rutile-type oxides.^106^ Using a third-order Birch–Murnaghan EoS^66^ and EoSFIT^107^ to fit the EoS parameters we determined *V*_0_ = 239.15(2) Å^3^, *B*_0_ = 251(1) GPa, and *B*_0_’ = 5.0(2). The bulk modulus agrees within 10% with the value we determined from elastic constants. This result makes AlWO_4_ not only the least compressible tungstate but also the least compressible AMO_4_ oxide,^1^ having a bulk modulus even larger than silicates and germanates.^108,109^ Interestingly, the bulk modulus of AlWO_4_ is four times that of Al_2_(WO_4_)_3_.^110^ This is because the first compound has a dense close-packed structure formed by incompressible AlO_6_ and WO_6_ octahedra, and the second one has an open and less dense structure where Al atoms are coordinated to six O atoms while the W atoms are coordinated to four O atoms.^110^

In a monoclinic crystal structure, the compressibility tensor is a symmetric tensor with four nonzero elements.^111^ Its eigenvalues are the compressibility along the principal axes of compression (the eigenvectors).^111^ We obtained them for AlWO_4_ from the results reported in Figure 11 using PASCAL.^112^ The main axes of compressibility are [010], [102], and [2̅01]. Their compressibilities are 0.99(1) 10^–3^, 1.10(1) 10^–3^, and 1.37(1) 10^–3^ GPa^–1^, respectively. The least compressible direction, [010], is the direction of the chains of AlO_6_ and WO_6_ octahedra. This is reasonable because compression along this direction can be achieved only by the reduction of the volume of the octahedra. In contrast, contraction in the other directions is also contributed by octahedral tilting.

From the phonon calculations we have obtained the frequencies of all Raman- and infrared-active phonons of AlWO_4_ as well as their pressure dependence, which is described by a quadratic polynomial. We summarize this information in Table S5 in the SI to facilitate phonon identification in future experiments. The total number of zone center phonon modes present is Γ_total_ = 10*A*_g_ + 8*B*_g_ + 8*A*_u_ + 10*B*_u_. Out of these, 2*A*_u_ and 1*B*_u_ are acoustic modes. The active optical modes are Γ_optical_ = 10*A*_g_ + 8*B*_g_ + 6*A*_u_ + 9*B*_u_, where *A*_g_ and *B*_g_ (*A*_u_ and *B*_u_) are Raman-active (infrared-active) modes.

We will present now results on the band structure of AlWO_4_ and the influence of pressure on it. The band structure and the electronic density of states, calculated using HSE06, are represented in Figure S6 in the SI and Figure 12. We found that AlWO_4_ is an indirect band gap semiconductor with a band gap energy of 2.29 eV. The top of the valence band is at a point of the Brillouin zone between Y_2_ and Γ, with coordinates (−0.2, −0.4, 0.4) in the reciprocal space. The bottom of the conduction band is at the L_2_ point of the Brillouin zone. As illustrated in Figure S6 (SI), two local maxima are also present in the valence band at L_2_ and V_2_, which are nearly equal in energy to the absolute maximum. On the other hand, in the conduction band, there are two local minima, at Γ and A, which are very close in energy to the absolute minimum. These conditions lead to a high density of states near the Fermi level, which can significantly enhance thermoelectric properties by increasing the thermopower of a material.^113^

**Figure 12:**
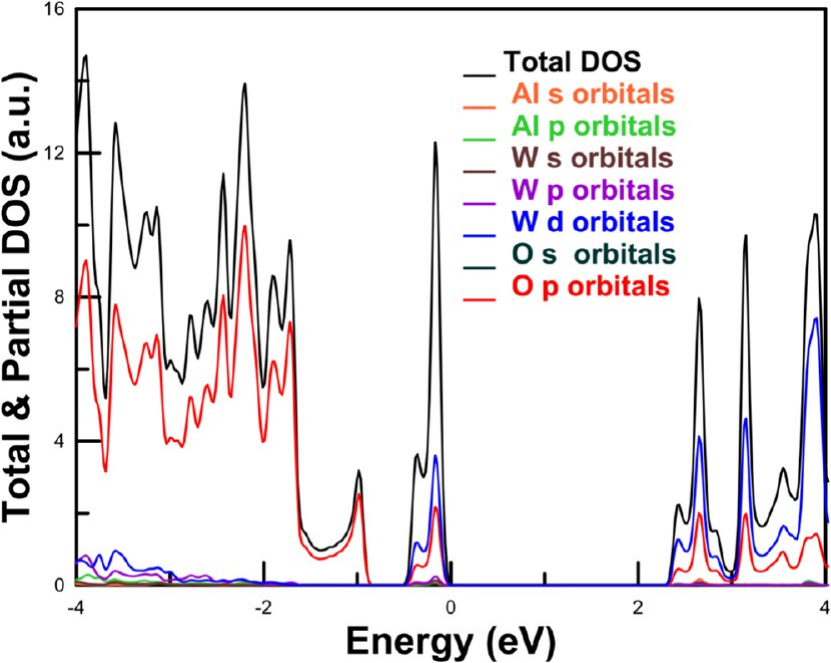
Electronic density of states of AlWO_4_ at 0 GPa.

There are no experimental values or prior calculations available for comparison with our results. We will compare it with Al_2_(WO_4_)_3_ and other AWO_4_ materials. The band gap of Al_2_(WO_4_)_3_ is 4.595 eV.^114^ We think that the smaller band gap of AlWO_4_ is related to the +5-oxidation state of W. Hexavalent tungsten oxides, such as WO_3_, generally have a larger band gap than pentavalent tungsten oxides because their W centers are in a + 6-oxidation state, and the conduction band is composed of empty W 5d orbitals. Pentavalent W^5+^ oxides have a smaller band gap as electrons populate the W 5d orbitals, leading to increased light absorption.^115^ In comparison to AWO_4_ compounds (refer to Table S2 in Supporting Information), we observe that the band gap of AlWO_4_ is comparable to that of CuWO_4_, CoWO_4_, FeWO_4_, and MnWO_4_, ranking among the tungstate compounds with lower band gap energy.

The analysis of the calculated DOS projected on atoms and orbitals presented in Figure 12 indicates that the uppermost levels in the valence band and the lowermost levels of the valence band consist mainly of O 2*p* and W 5*d* orbitals with a very small contribution of Al orbitals. Given that WO_6_ units exhibit incompressibility and considering that AlWO_4_ possesses a significantly higher bulk modulus in comparison to other AWO_4_ oxides, it is anticipated that the band gap energy of AlWO_4_ will be minimally influenced by pressure. We calculated the pressure dependence of the band gap energy and found it linearly increases with pressure from 2.29 eV at 0 GPa to 2.32 eV at 16.4 GPa. The pressure coefficient is 1.8 meV/GPa, which, as expected, is in absolute value smaller than all the pressure coefficients summarized in Table S2 in the SI.

### 6.3 CrWO_4_

CrWO_4_ is a material that shows potential for catalysis, particularly in the oxygen evolution reaction for water splitting,^116^ a vital step in water splitting for hydrogen production. This material has been identified as a semiconductor,^25^ but its band gap energy remains undetermined. CrWO_4_ has been reported to have two different crystal structures: an orthorhombic structure with eight formula units per unit cell (*Z* = 8) described by space group *F*222 ^26^ and a monoclinic structure with four formula unit per unit cell (*Z* = 4) described by space group *C*2/*m*.^25^ We have optimized both structures using DFT calculations and compare results to establish the most stable structure. In Figure S7 in the SI we report the total energy calculated for both structures as a function of the volume per formula unit. We found that the monoclinic structure is the one with the lowest total energy, supporting the structural assignment made by Vlasse et al.^25^ This structure is isostructural to the structure of AlWO_4_ and very similar to that of distorted rutile-type Cr_1–*x*_V_*x*_O_2_.^25^ In contrast with AlWO_4_, CrWO_4_ is magnetic.^117^ It undergoes an antiferro-paramagnetic transition at 45 K.^117^

The results from our calculations are in excellent agreement with experiments, as also happens with the case of AlWO_4_. The crystal structure optimized at 0 GPa exhibits the following unit-cell parameters: *a* = 9.3324 Å, *b* = 5.8298 Å, *c* = 4.6761 Å, and β = 91.85°. These values agree within 1% with the parameters obtained from X-ray diffraction experiments, which are *a* = 9.268 Å, *b* = 5.822 Å, *c* = 4.644 Å, and β = 91.90°.^25^

Through our DFT calculations, we have established the pressure dependence of the lattice parameters of CrWO_4_ up to 15 GPa. From these findings, we derive the pressure dependence of the unit-cell volume. The results are illustrated in Figure 13. We found that *a*, *b*, and *c* exhibit a comparable pressure dependence up to 5 GPa. We also found that the β angle decreases with pressure. Up to 5 GPa, the behavior of the crystal structure of CrWO_4_ is analogous to that of AlWO_4_. Beyond this pressure, the pressure dependence of the *a*- and *c*-axis starts to deviate from each other. The *c*-axis becomes less compressible as pressure increases, and the *a*-axis becomes more compressible. This phenomenon may be associated with alterations caused by pressure in the magnetic ordering of CrWO_4_, including the suppression of antiferromagnetism or the emergence of various magnetic ground states.^118^ It may also be linked to the occurrence of an isostructural phase transition.^119^ Both hypotheses warrant further investigation in future research.

**Figure 13:**
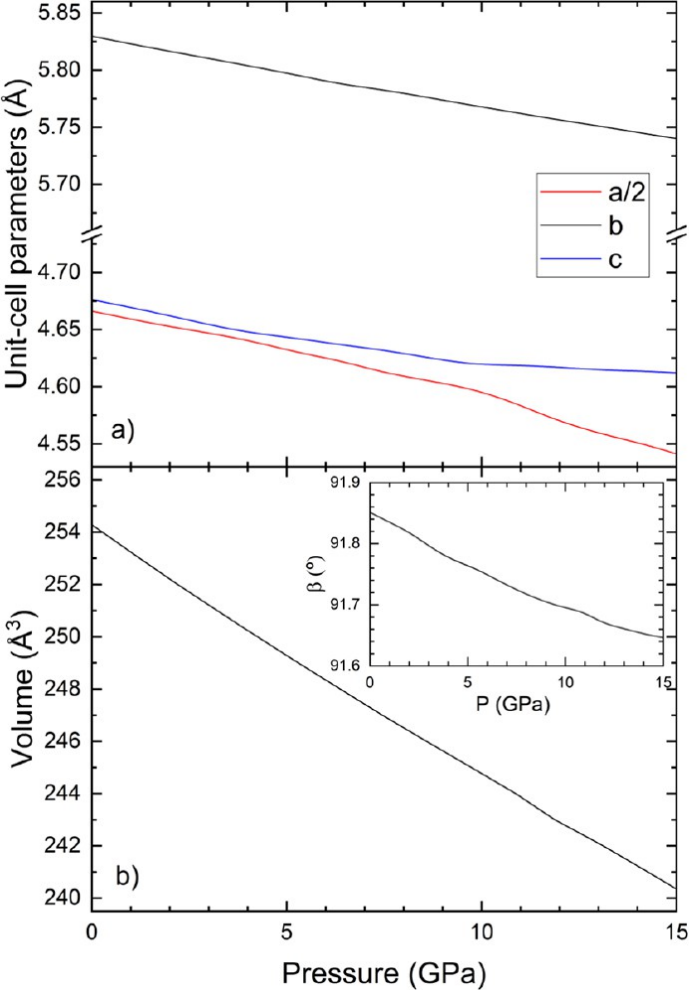
(a) Pressure dependence of unit-cell parameters of CrWO_4_. We plot *a*/2 to facilitate comparison with the other axes. (b) Pressure dependence of the unit-cell volume. The inset shows the pressure dependence of the β angle.

Concerning the relationship between volume and pressure, it is observed that from 0 to 15 GPa, the volume decreases by 5.6%, which makes CrWO_4_ as incompressible as AlWO_4_. By employing a third-order Birch–Murnaghan EoS^66^ and EoSFIT,^107^ we have established the following parameters *V*_0_ = 254.34(4) Å^3^, *B*_0_ = 239(1) GPa, and *B*_0_’ = 3.9(1). Using the method used for AlWO_4_, we have also calculated the main axes of compressibility of CrWO_4_ and the compressibility of each of them. The main axis of compressibility are [010], [101], and [2̅03]. Their compressibilities are 1.09(5) × 10^–3^, 1.19(5) × 10^–3^, and 1.58(4) × 10^–3^ GPa^–1^, respectively. The least compressible direction is [010] as in AlWO_4_.

We will now present the results regarding the electronic structure of CrWO_4_ and the impact of pressure on it. The band structure calculated using HSE06 is depicted in Figure 14. The calculated electronic density of states is shown in Figure S8 in the SI. As in AlWO_4_, both the conduction and valence bands have nearly degenerated extrema. CrWO_4_ is an indirect band gap semiconductor, exhibiting a band gap energy of 1.84 eV at 0 GPa (see Figure 14). There are two degenerated maxima in the valence band located at the Y_2_ and A points of the Brillouin zone. There are two degenerated minima in the conduction band, one at L_2_ point at the other near the Γ point. Therefore, CrWO_4_ could also be an efficient thermoelectric material.

**Figure 14:**
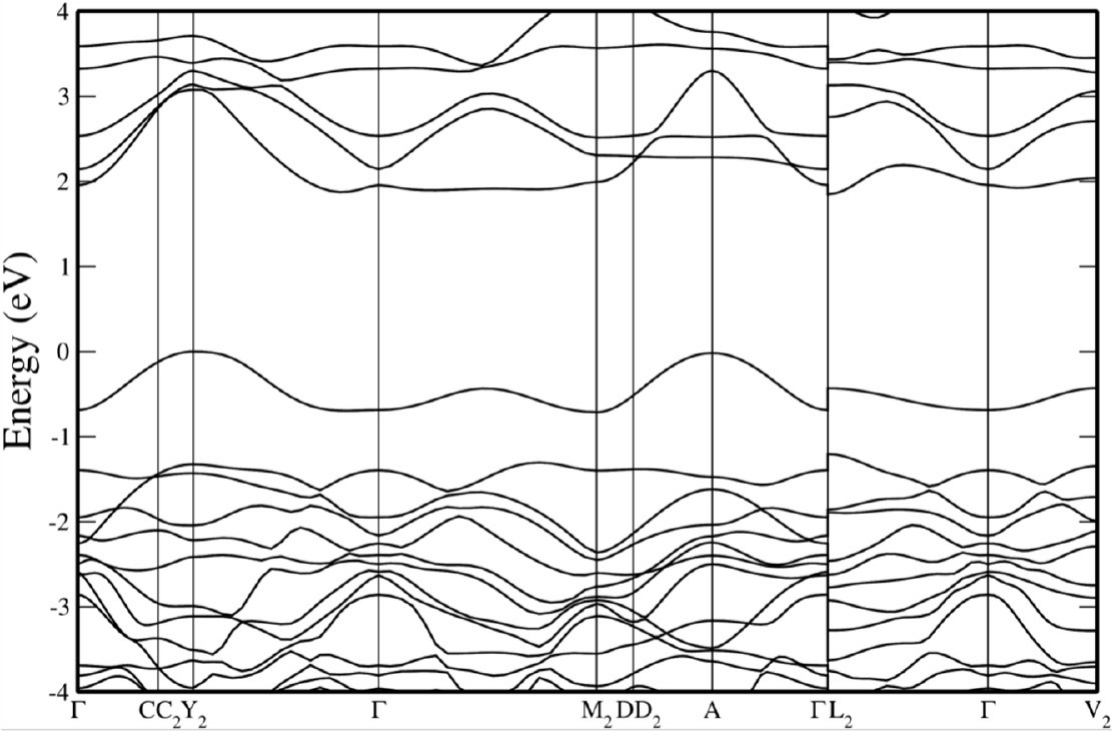
Band structure of CrWO_4_ calculated at 0 GPa using HSE06.

The density of states (DOS) projected onto atoms and orbitals is plotted in Figure S8 in Supporting Information. It reveals that the highest levels in the valence band are predominantly composed of O 2*p* orbitals, with a lesser contribution from W 5*d*. Conversely, the conduction band is primarily constituted of W 5*d* and O 2*p* orbital states, along with a contribution from Cr 3*d* orbitals. Regarding the pressure dependence of the band gap energy, we found that, as in AlWO_4_, in CrWO_4_, it is only slightly affected by pressure. We calculated the pressure dependence of the band gap energy and observed a linear increase with pressure, rising from 1.84 eV at 0 GPa to 1.92 eV at 15 GPa. The pressure coefficient was determined to be 5.3 meV/GPa.

### 6.4 HgWO_4_

It is known that at room temperature, HgWO_4_ does not undergo any structural phase transition up to 16 GPa.^56^ The influence of pressure in the crystal structure and Raman modes has already been characterized.^56^ However, the pressure dependence of the band gap energy has not been explored yet. Such studies are of particular interest because the related Hg_2_WO_4_ has been proposed as a candidate for pressure-driven metallization.^120^ Additionally, another aspect that heightens the interest in examining the electronic properties of HgWO_4_ is the fact that its band gap energy at 0 GPa has yet to be accurately determined. The literature presents band gap energies ranging from 3.9 to 4.2 eV.^121,122^ Nevertheless, the Materials Project database shows a calculated band gap of approximately 2.29 eV.^123^ This result was obtained from calculations that were performed with the GGA approximation, which incorporates the Hubbard correction. Here, we have calculated the band structure using GGA and the PBEsol functional and find that HgWO_4_ is an indirect semiconductor with a band gap energy of 2.10 eV. These findings are consistent with those documented in the Materials Project; however, it is recognized that both approximations, which are based upon the GGA, generally underestimate the band gap energy, which may hinder accurate predictions regarding pressure-induced metallization. More accurate calculations of the band structure are needed for studying high-pressure metallization. Thus, we also performed the band-structure calculations as a function of pressure using the hybrid HSE06 functional. With this method, we obtain a band gap energy of 3.50 eV, which is in better agreement with luminescence measurements (which report *E*_gap_ 3.9 eV^122^) than GGA calculations.

The calculated band structure is represented in Figure 15. The valence band has two degenerated maxima near the M_2_ point of the Brillouin zone. The minimum of the conduction band is at a point between the D_2_ and A points, but there are two other minima very close in energy, which are at the Γ and A points. Based on the computed electronic density of states, shown in Figure 16, in addition to the contributions from the O 2*p* and W 5*d* electronic states to the states close to the Fermi level, there is also a contribution from the Hg 6*s* and 5*d* orbitals. The top of the valence band is dominated by O 2*p* orbitals with a small contribution of Hg 6*s* and 5*d* states. The bottom of the conduction band is contributed equally by W, and Hg electrons, with a smaller contribution of O electrons. Thus, Hg 6*s* and 5*d* orbitals play in the electronic structure of HgWO_4_ the same role as Pb 6*s* and 5*d* orbitals play in PbWO_4_. Then the hybridization of O 2*p* states and W 5*d* states with the 6*s* and 5*d* states of Hg is expected to cause a reduction of the band gap under compression. Our findings confirm this hypothesis. The band gap of HgWO_4_ closes from 3.50 eV at 0 GPa to 2.79 eV at 15 GPa. The pressure coefficient is −47 meV/GPa, close to that of PbWO_4_, −62 meV/GPa. We also find that metallization is not expected to occur in HgWO_4_ in the range of stability of the low-pressure phase. An issue to explore in the future is if metallization might occur in the predicted HP phases,^56^ which are denser than the low-pressure phase and involve coordination changes for both cations.

**Figure 15:**
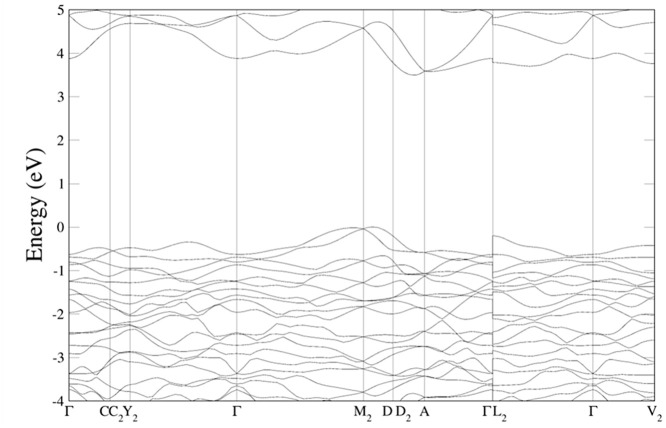
Band structure of HgWO_4_ calculated at 0 GPa using HSE06.

**Figure 16:**
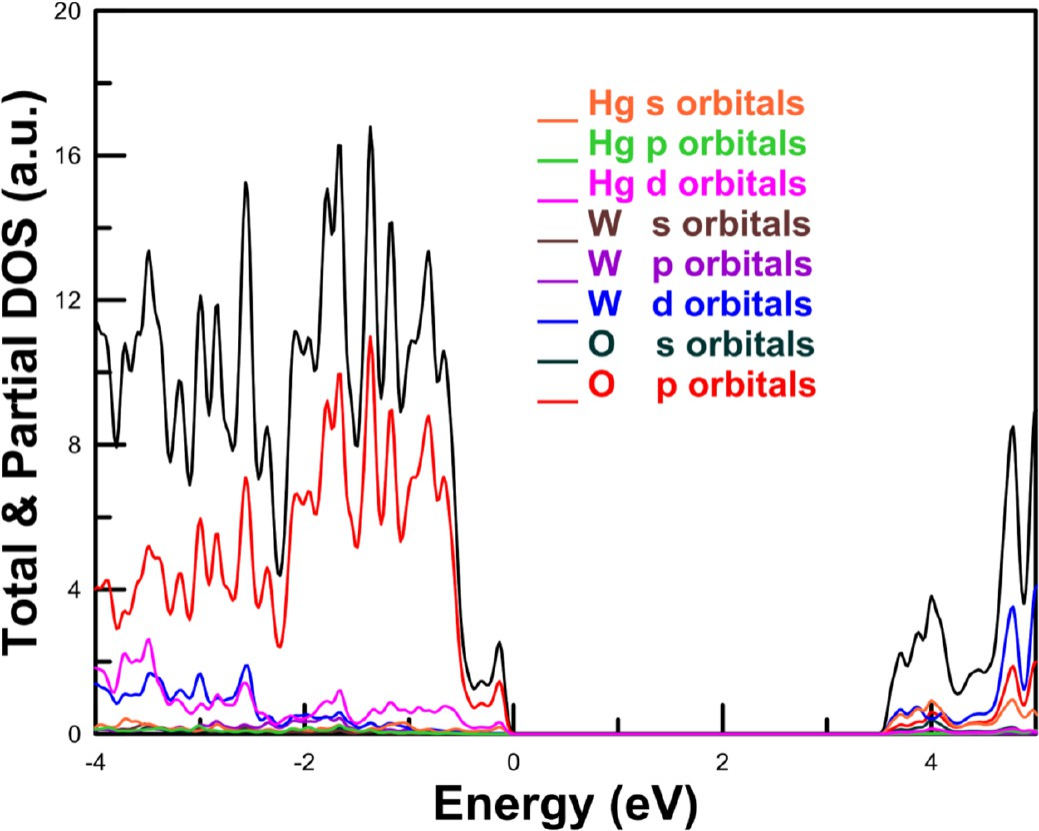
Electronic density of states of HgWO_4_ calculated at 0 GPa.

### 6.5 SnWO_4_

SnWO_4_ has been reported to absorb visible light due to the contribution of Sn 5*s*, which can be applied for solar water splitting reactions.^124^ As described previously in the article, this material has two different polytypes. The study of their electronic properties is crucial for water splitting applications. The two polymorphs have been studied under compression, showing that α-SnWO_4_ undergoes a phase transition at 15 GPa^57^ and β-SnWO_4_ decomposes at a similar pressure.^58^ The effect of pressure on the band structure has been studied by means of linear combination of atomic orbital (LCAO) calculations based on the hybrid exchange-correlation density functional Hartree–Fock scheme.^124,125^ These calculations predicted the metallization of SnWO_4_ around 15 GPa. This transition was assigned by an enhancement of the symmetry of metal–oxygen octahedra, which strengthens the interaction between Sn 5*s*, W 5*d*, and O 2*p* states, leading to the collapse of the band gap.^124^ The transitions between metal and insulator states in materials have garnered significant interest for over 50 years.^126,127^ This phenomenon has applications in innovative electronic and photonic devices, thereby encouraging the exploration of new functional materials.^128^ However, LCAO-based DFT underestimates band gaps due to the self-interaction error and the derivative discontinuity inherent in local and semilocal exchange-correlation functionals, which incorrectly raise occupied states and fail to accurately account for the difference in ground-state energies. Consequently, the metallization pressure might be underestimated. To address this issue, we have performed calculations of the pressure dependence of the band gap energy using the HSE06 functional.

The band structure and the electronic density of states calculated at 0 GPa are shown in Figures 17 and 18. For α-SnWO_4_ we obtain a band gap energy of 1.59 eV. The band gap is indirect, with the maximum of the valence band near the Γ point at (0,0,0.23) in the Brillouin zone. The bottom of the conduction band is at the Γ point. The band gap obtained with the HSE06 functional is very similar to the value of 1.52 eV reported by Harb et al. from similar calculations^129^ and to the value obtained from diffuse reflectance measurements^130^ of 1.62 eV. On the other hand, our band gap value is 0.15 eV larger than the value obtained with LCAO calculations, 1.45 eV. Notice that GGA + *U* calculations also underestimate the band gap energy yielding a value of 0.943 eV.^131^

**Figure 17:**
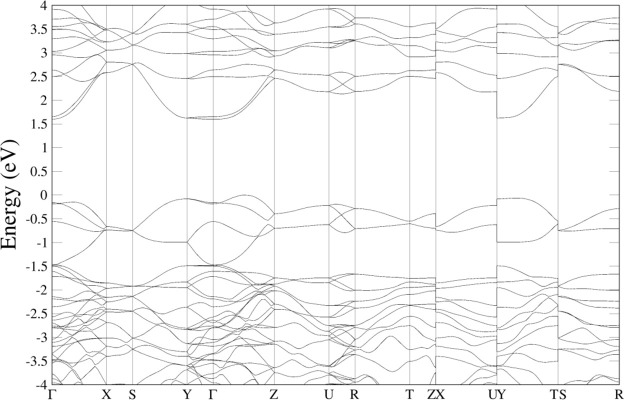
Band structure of α-SnWO_4_ calculated at 0 GPa using HSE06.

**Figure 18:**
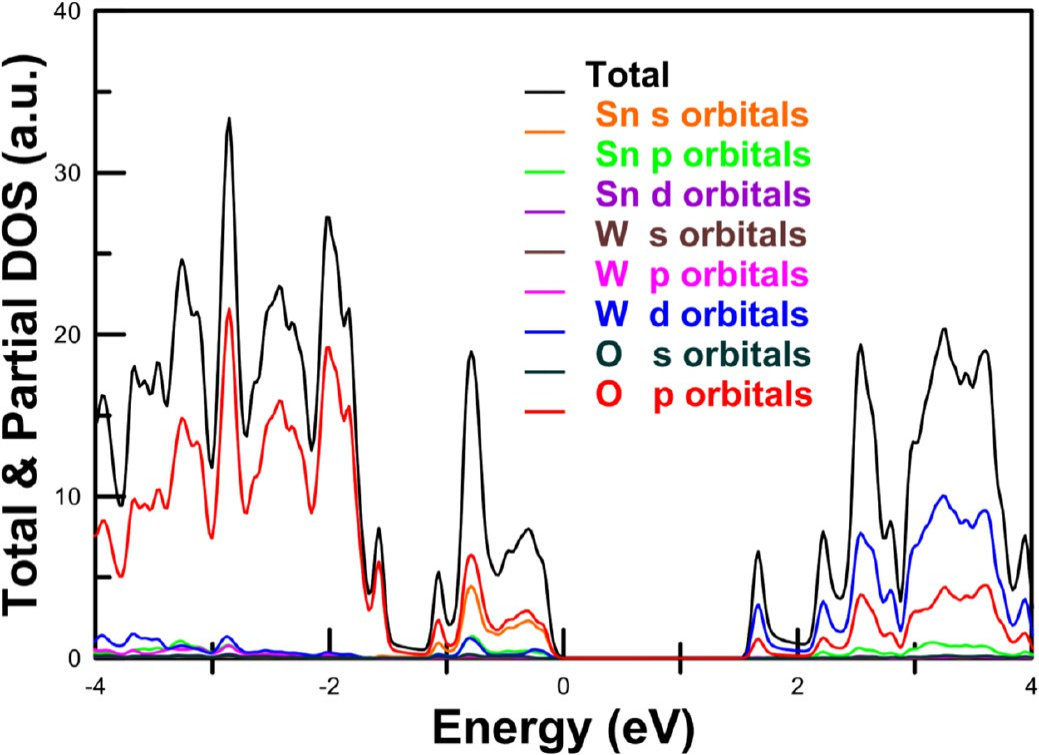
Electronic density of states of α-SnWO_4_ at 0 GPa.

According to the calculated electronic density of states shown in Figure 17, the O 2*p* and Sn 5*s* states primarily contribute to the upper portion of the valence band, with a minor inclusion of the Sn 5*p* and W 3*d* states. In contrast, the lower part of the conduction band is predominantly formed from the hybridized W 5*d*–O 2*p* states. The contribution of Sn 5*s* states to the bottom of the conduction band is enhanced under compression, which leads to a decrease of the band gap with pressure, similarly to what happens in PbWO_4_ and HgWO_4_. The band gap energy follows a nonlinear relation with pressure given by $E_{\mathrm{gap}}(P)=1.59\;\mathrm{eV}-0.096\;\frac{\mathrm{eV}}{\mathrm{GPa}}P+0.0017\;\mathrm{eV}/{\mathrm{GPa}}^{2}P^{2}$ , where *P* is in GPa. The calculated pressure coefficient at 0 GPa is −96 meV/GPa. Such coefficient is very similar to the one obtained from LCAO calculations.^124^ However, our results show that metallization does not occur up to the phase transition at 15 GPa. The band gap at 15 GPa is 0.50 eV. This means that metallization should occur in any of the high-pressure phases of SnWO_4_. This fact has not been studied yet and remains an open issue for future studies.

The computed band structure and electronic density of states for β-SnWO_4_ are shown in Figures S9 and S10 in the SI. As in α-SnWO_4_, in β-SnWO_4_ the valence band states below the Fermi level are formed by a strong mixing of fully filled Sn 5*s* and O 2*p* orbitals and a conduction band primarily consisting of empty W 5*d* orbitals. However, the calculated band gap is 4.46 eV, much larger than in α-SnWO_4_. This is because in β-SnWO_4_ the hybridization of orbitals is smaller than in α-SnWO_4_. Stronger hybridization between atomic orbitals leads to more delocalized electronic states, which generally decreases band gaps and increases the dispersion of the resulting hybridized bands. This is what occurs in SnWO_4_, as can be seen by comparing Figure 17 with Figure S9 (SI).

Our predicted bandgap value of 4.46 eV for the β-phase is found to be in excellent agreement with previous theoretical works (4.36 and 4.50 eV).^124,129^ On the contrary, existent GGA + *U* calculations underestimate the band gap energy reporting a value of 3.837 eV.^132^ According to the calculated band structure, β-SnWO_4_ is an indirect semiconductor. The top of the valence band is at (0, 0.3571,0) in the Γ-R direction of the Brillouin zone. The bottom of the conduction band is at (0.2727, 0.2727, 0) in the Γ-M direction of the Brillouin zone. The band gap energy of β-SnWO_4_ decreases in a similar fashion than in α-SnWO_4_. The pressure dependence of the band gap energy is described by $E_{\mathrm{gap}}(P)=4.46\;\mathrm{eV}-0.073\;\frac{\mathrm{eV}}{\mathrm{GPa}}P+0.0025\;\mathrm{eV}/{\mathrm{GPa}}^{2}P^{2}$ , where *P* is in GPa. The pressure coefficient at 0 GPa is −73 meV/GPa.

## 7 Future Perspectives

In this section, we will explore the various future opportunities that high pressure applied to tungstates can provide. One interesting subject is pressure-driven amorphization in scheelite-type AWO_4_ tungstates. As described in Section 3, amorphization was observed in CaWO_4_, BaWO_4_, and PbWO_4_ at pressures between 35 and 50 GPa. High-pressure amorphization is important for creating new metastable materials with unique properties not achievable through conventional means, such as enhanced ionic conductivity in batteries, new high-density phases, and altered electrical or mechanical properties.^133^ The three most interesting problems to explore in the future are (1) Does pressure-induced amorphization also occur in EuWO_4_ and SrWO_4_ tungstates? (2) What is the mechanism behind amorphization? (3) Is amorphization of scheelite inherent to compression or is it caused by nonhydrostatic stresses?

An additional area of interest for upcoming research is the expansion of pressures investigated in wolframites to 50 GPa. It is expected that all wolframites would undergo phase transitions, not reported yet, between 20 and 50 GPa, as NiWO_4_ undergoes at 27 GPa.^7^ A case of significant interest is FeWO_4_. This material is a very promising candidate for an effective photocatalyst in water purification applications.^134^ FeWO_4_ is the wolframite with the smallest band gap, 2.0 eV,^8^ and it should exhibit the lowest metallization pressure among wolframites. High-pressure metallization, a critical area of high-pressure research, helps understand conductivity mechanisms and discover new states of matter.^135^

It would also be of great interest to advance in the future in the study of the effects of pressure on the magnetic properties of wolframites like CoWO_4_, FeWO_4_, MnWO_4_, and NiWO_4_. Up to now, only two studies have been performed in this subject. MnWO_4_ has been studied up to temperatures of 7.8 K, but with pressure limited to 1.7 GPa.^136^ The low-temperature commensurate and paraelectric phase were stabilized under compression, and the stability range of the ferroelectric phase was diminished under pressure.^136^ The temperature dependence of the magnetic properties of FeWO_4_ was studied by high-pressure neutron diffraction^51^ up to a maximum pressure of 8.7 GPa and a minimum temperature of 30 K. It was discovered that, even though a contraction of 5% in volume was produced, the maximum pressure applied in this study had a slight effect on the orientation of magnetic moments and the Néel temperature. Studying magnetism under pressure provides insight into its relationship with other properties like structural transitions and electronic states, which is vital for understanding and designing functional materials.^137^ The expansion of magnetic research on wolframites to greater pressures than those addressed by the few existing high-pressure magnetic studies could unveil intriguing research opportunities. In particular, the investigation of materials exhibiting both magnetic and electric order within the same phase is particularly fascinating; however, the scientific community has dedicated minimal efforts to this area.^138^

Future studies on AWO_4_ compounds under high pressure should also encompass AlWO_4_ and CrWO_4_. Specifically, it would be intriguing to verify through experiments in diamond-anvil cells whether these compounds are as incompressible as our calculations suggest. In the case of CrWO_4_, it would also be of significant interest to investigate what happens to its magnetic properties under pressure. Furthermore, another area that remains to be experimentally explored is the effect of pressure on the electronic properties of HgWO_4_ and SnWO_4_. For both compounds, we predict the pressure dependence of the band gap energy in this work. The latter is a compelling candidate for examining the potential metallization of either of its two high-pressure phases. Regarding HgWO_4_, it would also be pertinent to study it experimentally at pressures exceeding those investigated thus far to test the theoretical predictions of phase transitions above 20 GPa.^56^

It has been reported that single crystals of PbWO_4_ produced using the Czochralski method in the presence of PbO deficiency in the melt, crystallize in a tetragonal structure which is different than scheelite.^139^ This structure is described by space group *P*4/*nnc*.^139^ Oxygen and lead sites are not fully occupied. The formula of the obtained compound is Pb_7_W_8_O_28.8_. Vacancies are known to play a significant role in the high-pressure behavior of solids by affecting the compressibility of a material and phase transitions.^140^ High pressure can stabilize specific vacancy-related defect structures. This can lead to changes in material properties, such as altering the bulk modulus or inducing new structural phases. Therefore, future high-pressure studies on compounds like Pb_7_W_8_O_28.8_ could lead to unexpected findings.

A promising avenue for future research involves germanium tungstate (GeWO_4_), a material distinguished by its unique combination of structural, optical, and electronic properties. These features make GeWO_4_ an attractive candidate for interdisciplinary exploration, bridging materials science, photonics, and energy technologies.^141^ As a tungstate compound, GeWO_4_ exhibits promising photoluminescence, catalytic behavior, and potential as a scintillator material, making it relevant for applications in medical imaging, radiation detection, and environmental sensing.^141^ Furthermore, the presence of germanium introduces opportunities to explore tunable band gaps, nonlinear optical effects, and structural versatility under varying pressure. Up to now, GeWO_4_ has never been studied under compression. GeWO_4_ and germanium deficient Ge_0.8_WO_4_ adopts the monoclinic wolframites-type structure in the space group *P2/c*. The structure is characterized by highly distorted WO_6_ and GeO_6_ octahedral units forming infinite edge sharing zigzag chains that run parallel to the *c*-axis. Compared to GeWO_4_, the *Ge* vacancies in Ge_0.8_WO_4_ narrow the band gap and shift the absorption edge to a lower energy.^141^ This phenomenon might favor pressure-driven metallization. They also might trigger structural instabilities at lower pressures than in the rest of wolframites, favoring phase transitions below 20 GPa. According to the discussion presented in Section 4.2 of this article, GeWO_4_ is expected to have bulk modulus like that of other wolframites, with an estimated value of 145(15) GPa.

Additionally, it would be interesting also to extend the high-pressure studies to tugstates with compositions different than AWO_4_. One case of interest is In_2_W_3_O_12_, a compound that exhibits uniaxial negative thermal expansion, and overall positive volume expansion.^142^ This compound was reported to undergo phase transitions at 1.9 and 2.7 GPa.^142^ The phase transition is associated with a collapse in compressibility, leading to an increased compressibility in the denser high-pressure polymorph. This phenomenon resembles findings in other compounds within the Sc_2_W_3_O_12_ family.^143^ The cause of this fascinating behavior remains uncertain and requires additional experimental and theoretical investigation. High-pressure single-crystal XRD could contribute to the understanding of the mechanism of the phase transition and the causes leading to the anomalous increase of compressibility.

Sc_2_(WO_4_)_3_ undergoes reversible phase transitions at 0.6 and 1.6 GPa. At 6.5 GPa it was detected the emergence of a disordered crystalline state which evolves into an amorphous phase beyond 14 GPa.^143^ Al_2_(WO_4_)_3_ suffers two reversible phase transitions at pressures below 3 GPa. In addition, it undergoes two more phase transitions at 5.3 and 6 GPa before transforming irreversibly to an amorphous phase at 14 GPa.^144^ However, the determination of the structure of the high-pressure phase found above 5.3 GPa is a task that remains open. Another subject that deserves to be explored is the relationship between crystalline disorder and amorphization with nonhydrostatic stresses. Only XRD experiments loading diamond-anvil cells using helium or neon as pressure medium will clarify if amorphization is inherent to compression of Sc_2_(WO_4_)_3_ and Al_2_(WO_4_)_3_ or it might be triggered by nonhydrostatic stresses.

To further extend the study of orthotungstates, it is worth considering how high-pressure studies could also probe the underlying mechanisms of phase transitions in Li_2_WO_4_, Na_2_WO_4_, and other alkali-metal tungstates^145,146^ potentially revealing new polymorphs or metastable phases that may exhibit novel properties. The transformation from tetrahedral to octahedral coordination, for instance, could alter the electronic band structure significantly, potentially giving rise to new electronic or magnetic phases that are not observed at ambient pressure. This is particularly intriguing because the coordination environment around the tungsten atom plays a pivotal role in determining the material’s electronic properties. As the pressure increases, changes in the bonding network could lead to modifications in the material’s electronic band gap, which might be tuned for specific applications, such as in optoelectronics or sensors.

Additionally, examining these alkali-metal tungstates under high pressure could provide valuable insights into their ionic conduction properties. High-pressure conditions may induce changes in the ionic pathways or facilitate more efficient ion transport, especially for Li^+^ and Na^+^ ions, which are crucial for applications as solid-state batteries. Given that ionic conductivity is highly sensitive to the crystal structure, it is possible that high-pressure conditions could create more favorable diffusion pathways for lithium or sodium ions, enhancing the performance of these compounds as solid electrolytes. Moreover, exploring the behavior of Li_2_WO_4_ and Na_2_WO_4_ under high pressure could reveal a relationship between pressure-induced structural modifications^145,146^ and the ionic conductivity of these materials, offering a pathway to optimize materials for next-generation battery technologies.

## 8 Conclusions

The application of high pressure can lead to a range of novel and intriguing phenomena, often revealing unexpected and remarkable behaviors in materials. In the case of AWO_4_ orthotungstates, structural phase transitions have been induced by pressure in various tungstates, unveiling new material properties that are not apparent under ambient conditions. These high-pressure-induced phase transitions provide valuable insight into the underlying atomic and electronic structures of these compounds.

One of the most remarkable examples of such behavior is found in scheelites, which undergo phase transitions below 10 GPa, followed by a process of amorphization beyond 35 GPa. This transformation is notable not only for the dramatic change in structural integrity, but also for the loss of crystallinity as the material undergoes irreversible changes under extreme conditions. Such phenomena highlight the complexity of the structural evolution of tungstates under pressure and underscore the need for a deeper understanding of the mechanisms driving these transformations.

A particularly striking phenomenon is the variation in the high-pressure behavior of the electronic bandgap, which has been observed to differ significantly across various tungstate compounds. In materials like PbWO_4_ and SnWO_4_, changes in the bandgap can exceed 1 eV within the pressure range covered by current studies. This phenomenon is crucial for understanding how pressure can influence the electronic properties of materials, with potential implications for optoelectronic and photonic applications. The pressure-induced changes in the electronic structure can lead to new opportunities for designing materials with tunable electronic properties, which are vital for developing devices such as sensors, light-emitting diodes, and even quantum computing components.

This article presents a comprehensive review of recent findings related to both scheelite- and wolframite-type tungstates, along with other AWO_4_ compounds. It details their phase transitions, including the transformation mechanisms and the high-pressure behavior of their structural and electronic properties. A systematic analysis of the reported results is provided, focusing on how pressure can modify the characteristics of these materials, offering potential pathways for their integration into cutting-edge technological applications. By consolidating the findings from a range of experimental and computational studies, we aim to provide a clearer understanding of the pressure-induced phenomena that govern the properties of tungstates.

In addition, we have presented new predictions based on density-functional theory calculations performed for previously uncharted compounds, such as BeWO_4_, AlWO_4_, and CrWO_4_. These compounds, while not yet experimentally studied at high pressure, show promising potential for discovering novel pressure-induced properties. Our theoretical insights offer a starting point for experimental investigations into these compounds, which could lead to the identification of new high-pressure phases with unique characteristics. The inclusion of these lesser-studied compounds enriches our understanding of the broader behavior of AWO_4_ tungstates under extreme conditions.

Unaddressed yet promising research issues are also discussed in this work. Key challenges remain in the comprehensive understanding of the relationships between structure and electronic properties under high pressure. For instance, the mechanisms behind amorphization in certain tungstates, as well as the role of pressure in tuning material properties such as superconductivity, magnetism, and thermoelectric efficiency, require further exploration. Additionally, the development of experimental techniques to observe high-pressure phases in real time remains a critical area for improvement.

We dedicate a section to exploring these unsolved problems and suggest future research avenues that could lead to new discoveries in the field of high-pressure physics and materials science. In particular, we highlight the potential for interdisciplinary research combining advanced experimental techniques with cutting-edge computational methods to unlock the full range of pressure-induced phenomena in tungstates. We hope that this article will inspire future studies that ultimately will lead to exciting advancements in both fundamental physics and practical applications, offering new insights into the design of materials with tailored properties for a wide range of technological fields. The study of high-pressure behavior in tungstates has the potential to push the boundaries of materials science and open up new avenues for the development of next-generation electronic, photonic, and energy-harvesting technologies. By advancing our understanding of pressure-induced phase transitions and electronic structure modifications, we may be able to engineer tungstate-based materials with enhanced functionalities that are critical for emerging applications in sustainable energy solutions.

## Supplementary Material



## Data Availability

The data that support the findings of this study are available from the corresponding author upon reasonable request.

## Notes

XRD patterns, data from the literature, calculated phonon dispersion curves, and calculated band structures and electronic density of states (PDF)
