# Supplementary material for: Insights into the High-Pressure Behavior of AWO4‑Type Orthotungstates
Source: J Phys Chem C Nanomater Interfaces. 2025 Dec 26;130(9):3201–25. doi: 10.1021/acs.jpcc.5c07394 (PMC12970149; doi:10.1021/acs.jpcc.5c07394)
Supplement: Supplementary file 1 [file jp5c07394_si_001.pdf]

Supporting Information

Insights into the high-pressure behavior of AWO_4-type orthotungstates

Alfonso Muñoz[1], Silvana Radescu[2], Andrés Mujica[2], Daniel Errandonea[3,*]

[1]Departamento de Física, MALTA Consolider Team, Universidad de La Laguna, San Cristóbal de La Laguna, Tenerife E-38200, Spain

[2]Departamento de Física, MALTA-Consolider Team, Instituto de Materiales y Nanotecnología, Universidad de La Laguna, San Cristóbal de La Laguna, E-38200 Tenerife, Spain

[2]Departamento de Física Aplicada - Instituto de Ciencia de Materiales, MALTA Consolider Team, Universidad de Valencia, Edificio de Investigación, C/Dr Moliner 50, 46100 Burjassot, Valencia Spain

*daniel.errandonea@uv.es

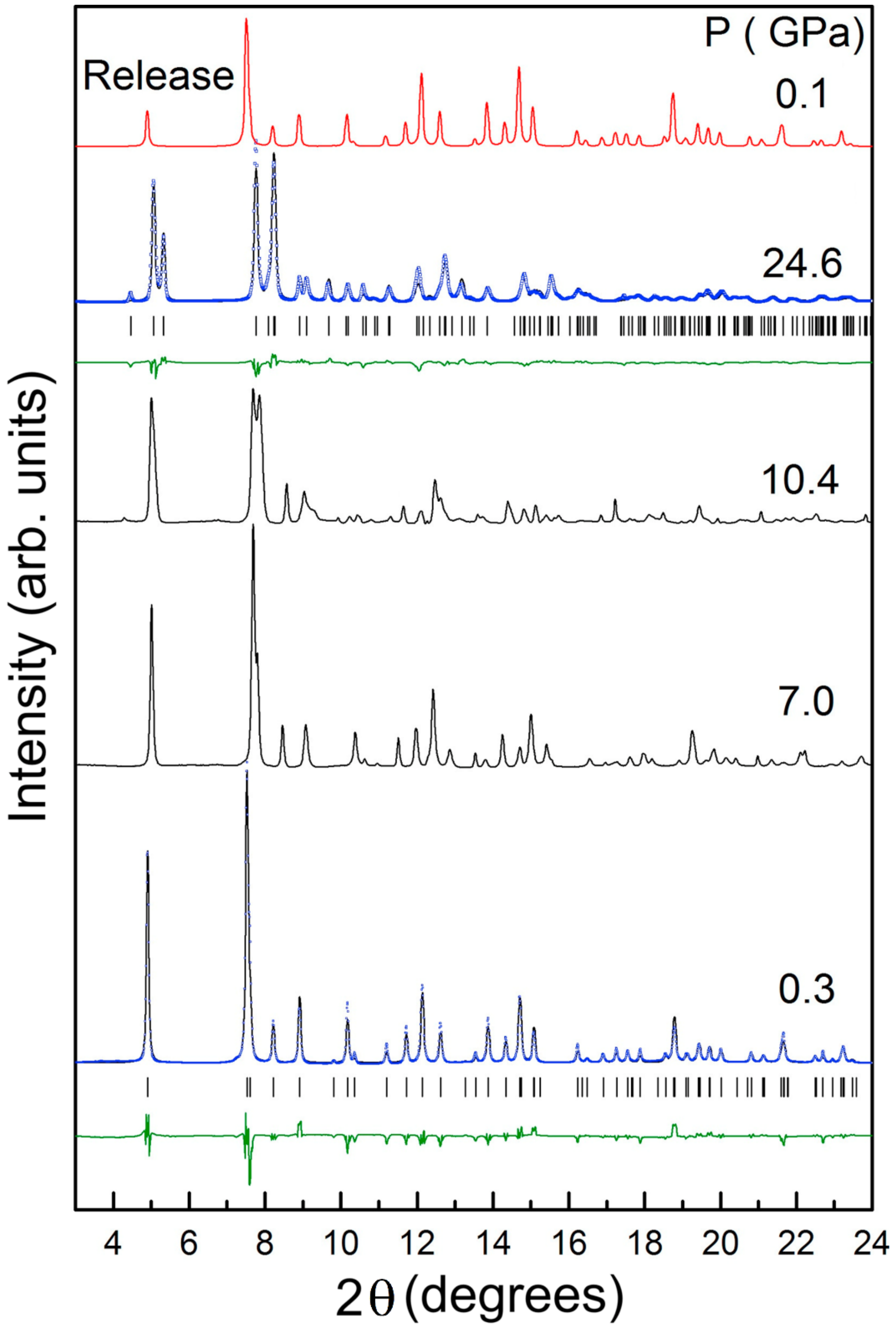

Figure S1: XRD patterns measured in $CaWO_4$ at different pressures measured by this group and previously reported by Vilaplana *et al.*[S1] Copyright 2014 Elsevier. The pressures are indicated in the plot. λ = 0.40695 Å. Rietveld refinements are shown for the scheelite structure at 0.3 GPa and for the HP fergusonite phase at 24.6 GPa. At both pressures experiments are shown as symbols, refinements as blue lines, and residuals as green lines. Ticks indicate the position of Bragg reflections. In red, we show a XRD pattern measured after decompression, showing the reversibility of the transition.

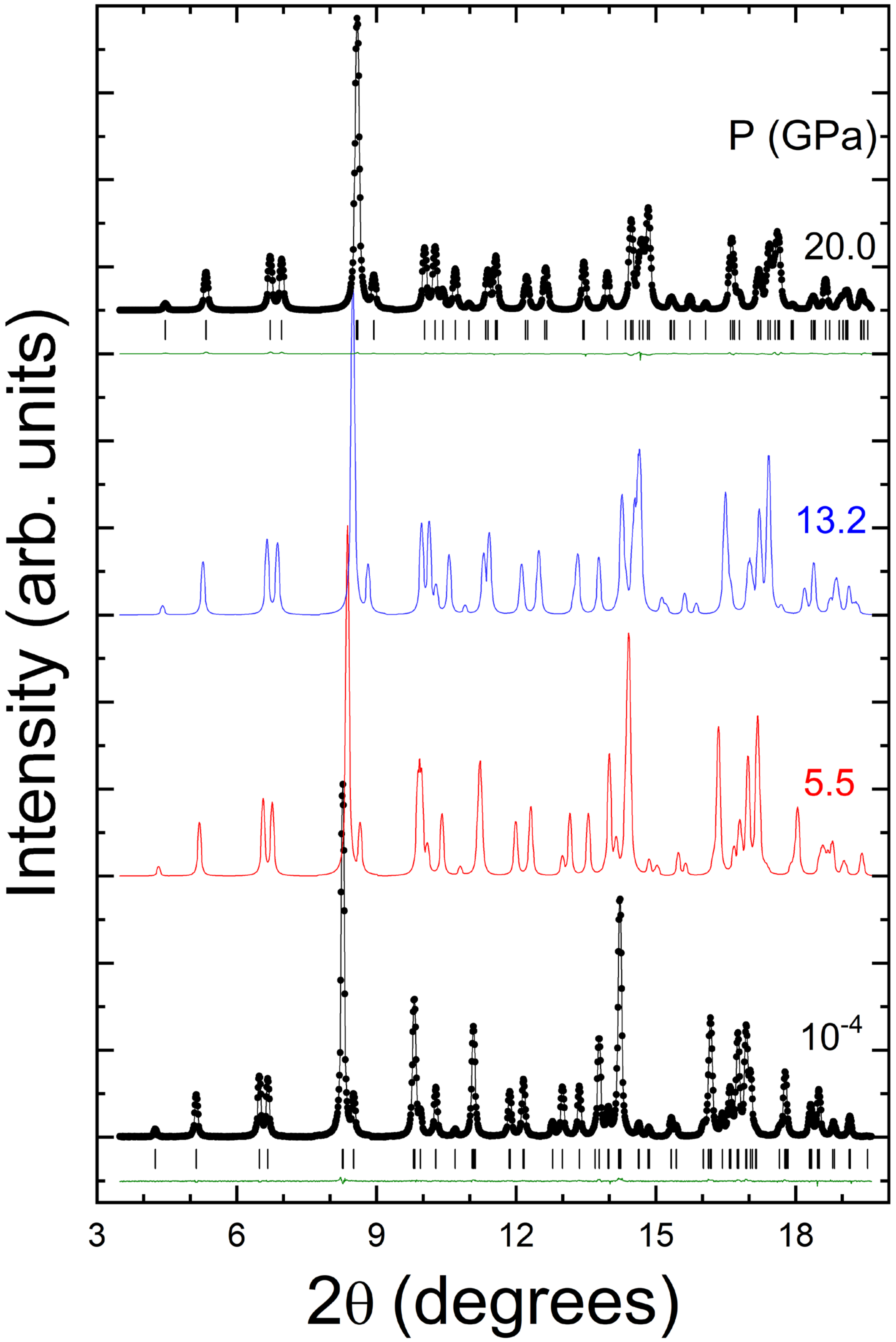

Figure S2: XRD patterns measured by our group in $FeWO_4$ at different pressures which are indicated in the figure. The figure is adapted from Ref. S2. Copyright 2024 American Chemical Society. λ = 0.4642 Å. Rietveld refinements are shown for the wolframite structure at 10^{-4} GPa and 20 GPa. At these pressures, experiments are shown as symbols, refinements as lines, and residuals as green lines. Ticks indicate the position of Bragg reflections.

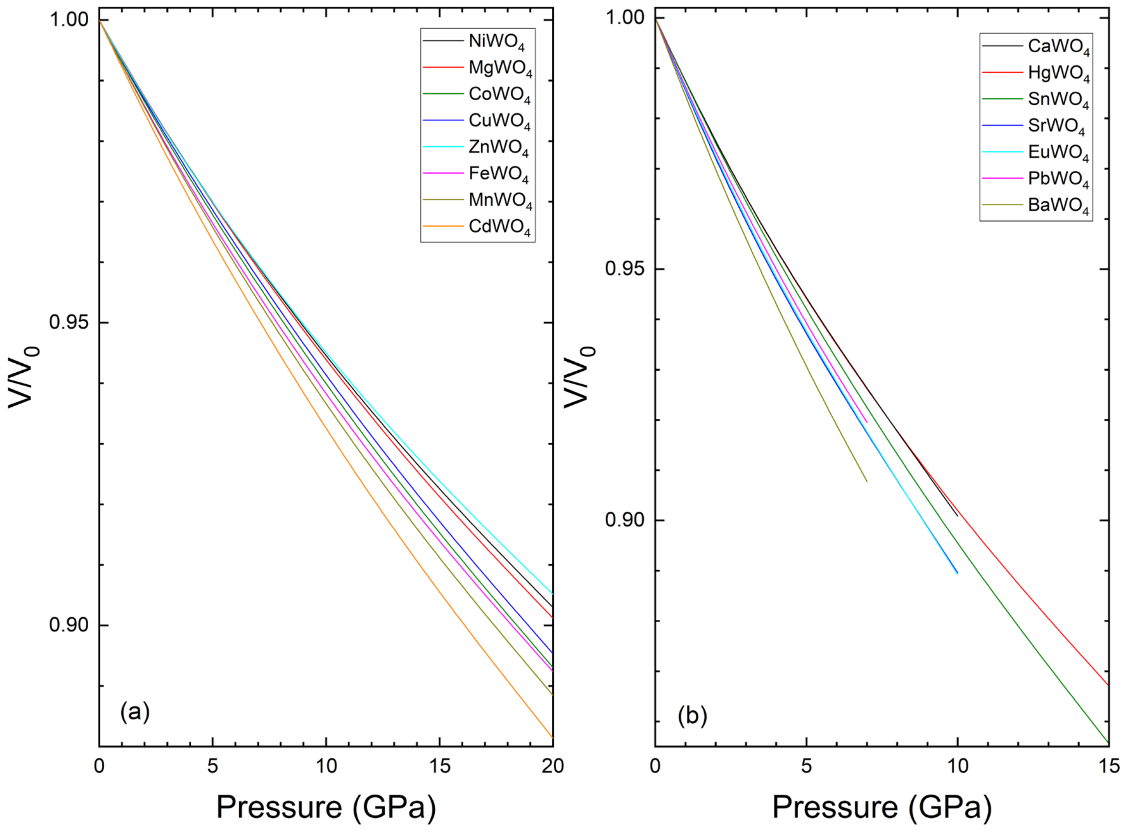

Figure S3: Relative change of normalized unit-cell volumes (V/V0) with pressure. The different lines are identified in the figure. They described results from the literature using the equations of state summarized in **Table S1**. (a) Results for $NiWO_4$, $MgWO_4$, $CoWO_4$, $CuWO_4$, $ZnWO_4$, $FeWO_4$, $MnWO_4$, and $CdWO_4$. (b) Results for $CaWO_4$, $HgWO_4$, $SnWO_4$, $SrWO_4$, $EuWO_4$, $PbWO_4$, and $BaWO_4$.

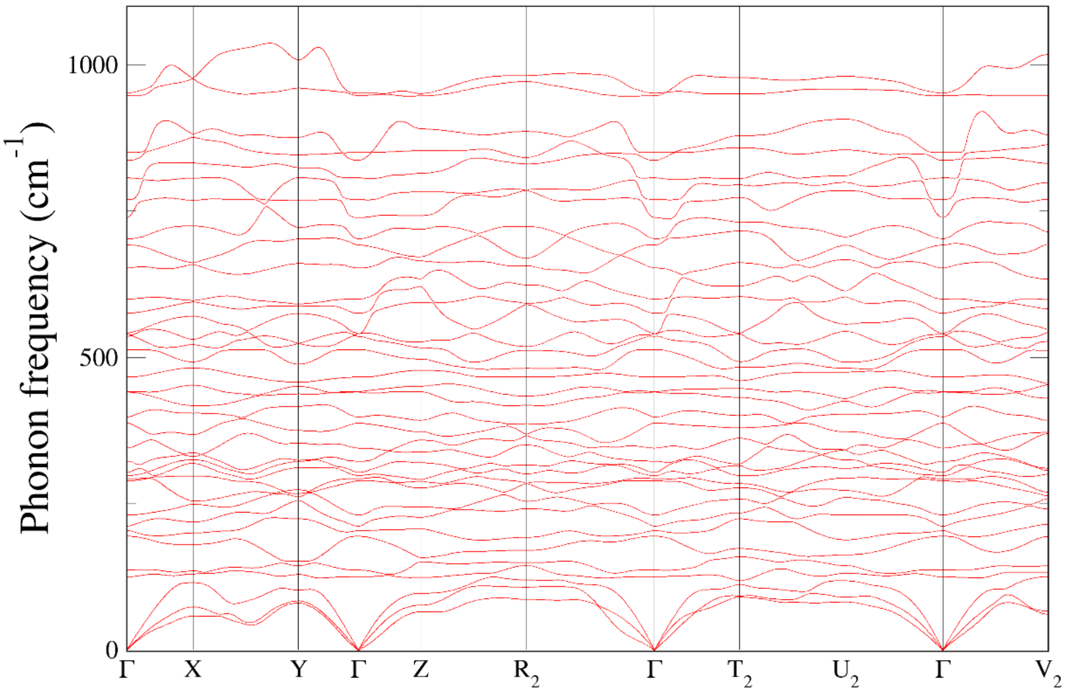

Figure S4: Calculated phonon dispersion of triclinic $BeWO_4$ at 0 GPa.

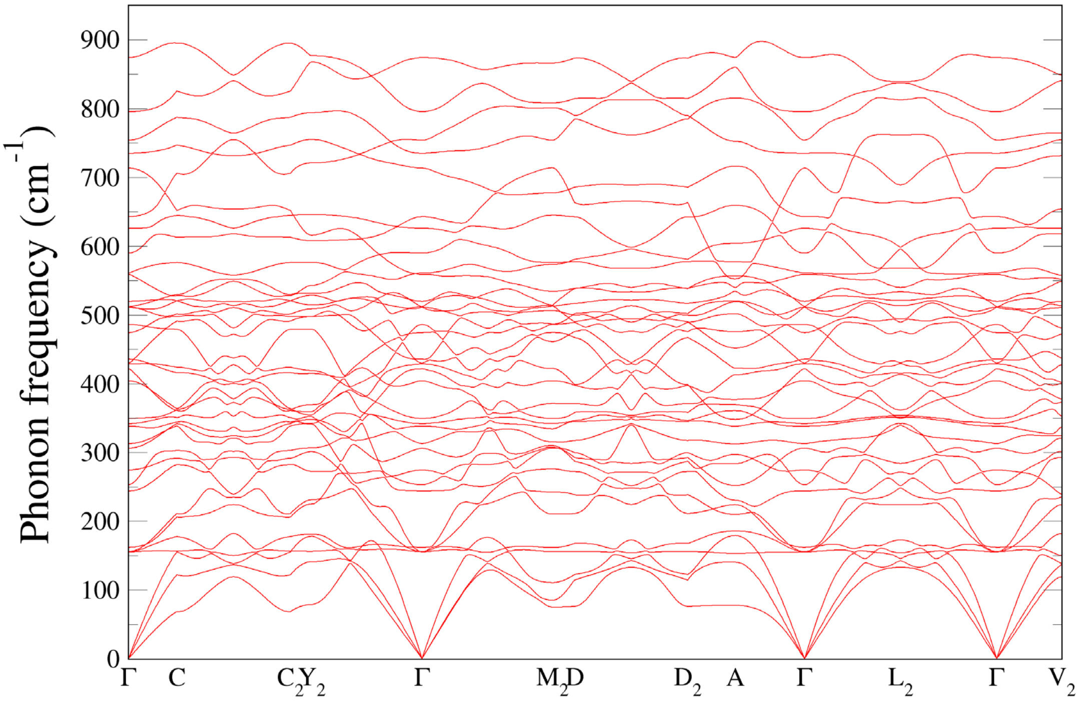

Figure S5: Calculated phonon dispersion of $AlWO_4$ at 0 GPa.

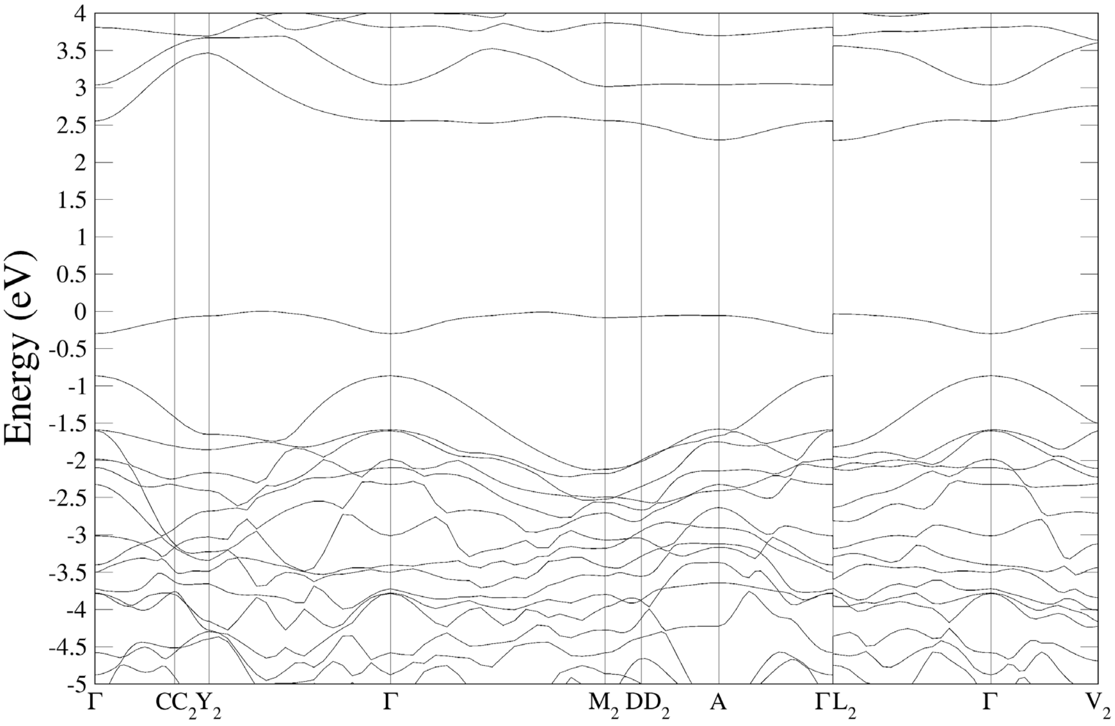

Figure S6: Band structure of $AlWO_4$ calculated at 0 GPa using HSE06.

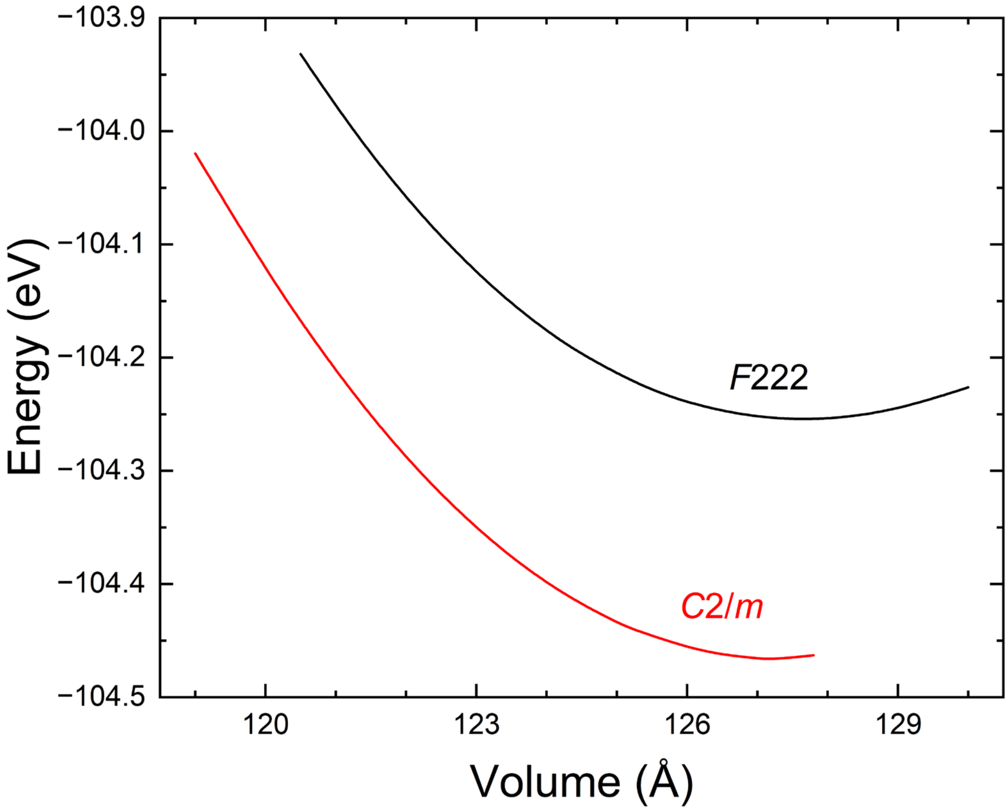

Figure S7: Total energy versus volume calculated for the two structures of $CrWO_4$ reported in the literature. The structures are identified by the space group symbol. The results of the orthorhombic structure were renormalized Z = 4 for comparison.

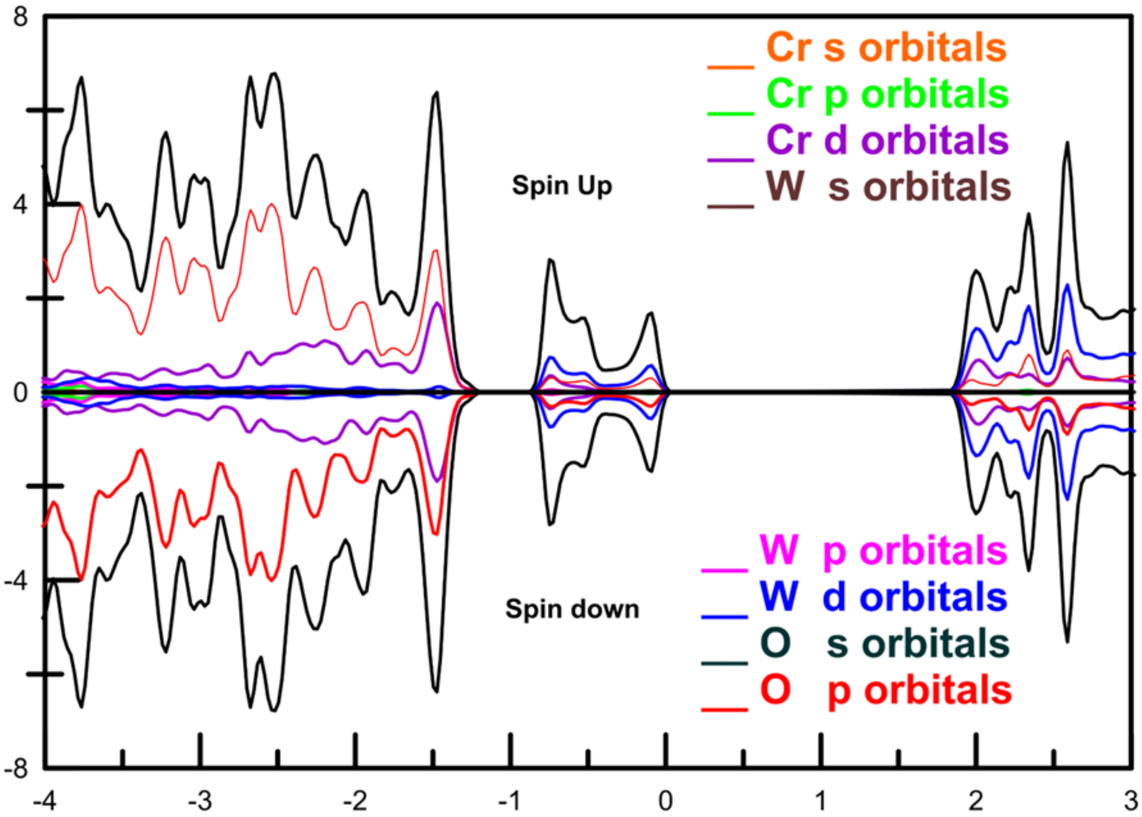

Figure S8: Spin-polarized electronic density of states of $CrWO_4$ calculated at 0 GPa.

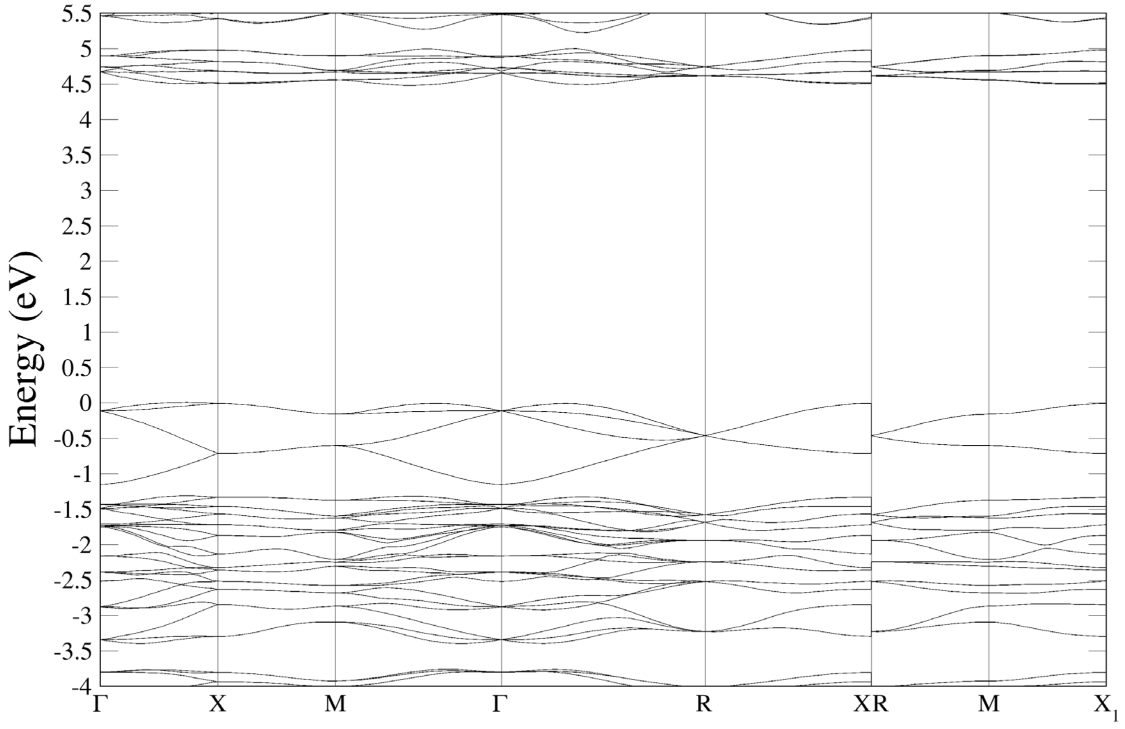

Figure S9: Band structure of β-$SnWO_4$ calculated at 0 GPa using HSE06.

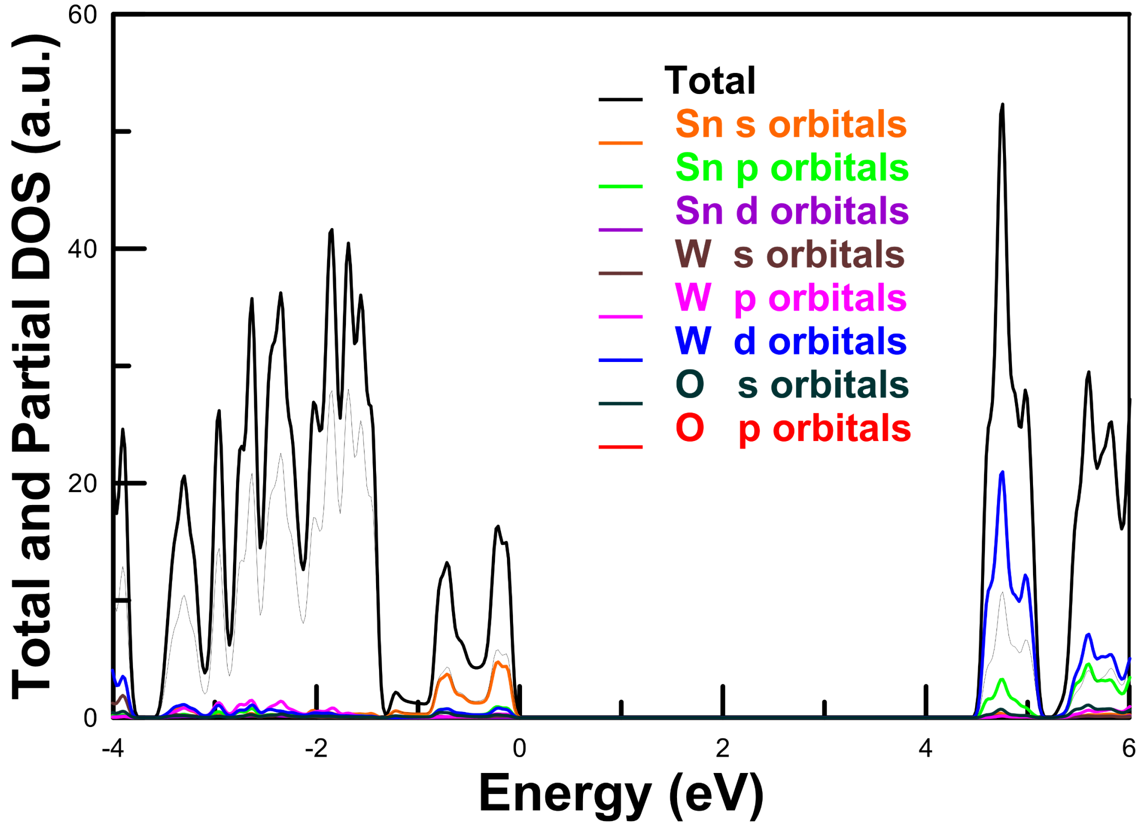

Figure S10: Electronic density of states of β-$SnWO_4$ calculated at 0 GPa.

Compound	V_0 (Å^3)/Z	B_0 (GPa)	B_0'	References
$NiWO_4$	64.6(1)	148(9)	5.5(3)	[S3]
$MgWO_4$	65.6(1)	152(8)	4.5(3)	[S4]
$CoWO_4$	65.7(1)	141(10)	4	[S5]
$CuWO_4$	66.4(3)	145(9)	4	[S6, S7]
$ZnWO_4$	66.5(3)	145(6)	6.6(9)	[S4]
$FeWO_4$	67.5(3)	136(2)	5.0(4)	[S2]
$MnWO_4$	69.4(1)	131(2)	4.3(2)	[S8]
$CdWO_4$	74.7(1)	123(2)	4	[S9]
$CaWO_4$	78.0(3)	73(5)	5.2(4)	[S10, S11, S12, S13]
$HgWO_4$	80.9(3)	73(5)	5.1(4)	[S14]
$SnWO_4$	81.9(3)	74(6)	3.4(1.5)	[S15]
$SrWO_4$	86.9(3)	63(7)	5.2(9)	[S10]
$EuWO_4$	87.2(2)	65(6)	4.6(9)	[S16]
$PbWO_4$	89.5(2)	66(5)	5.6(9)	[S17]
$BaWO_4$	100.9(2)	59(7)	4.2(8)	[S17, S18, S19]

Table S1: The equation of states parameters, Volume (per formula unit) at zero pressure (V_0), bulk modulus at zero pressure (B_0), and its pressure derivative (B_0'), determined from different studies. Here we quote average values and indicate the standard deviation between brackets. The literature references from which the parameter values were taken are indicated in the table.

Compound	E_{gap} (eV)	Type	VBM	CBM	dE_{gap}/dP (meV/cm^{-1})	References
$NiWO_4$	3.00(5)	Indirect	Γ-A	Y2	-13(1)	[S3, S20]
$MgWO_4$	4.06(5)	Direct	Z	Z	13.9(5)	[S21]
$CoWO_4$	2.25(1)	Direct	Γ	Γ	-28.1(4)	[S5]
$CuWO_4$	2.30(5)	Indirect	X	Y	-11.0(5)	[S22]
$ZnWO_4$	3.98(5)	Direct	Z	Z	12.7(5)	[S21]
$FeWO_4$	2.00(5)	Indirect	Y	Γ	-25.0(5)	[S2]
$MnWO_4$	2.37(5)	Indirect	D	Z	-22.2(5)	[S21]
$CdWO_4$	4.02(5)	Direct	Z	Z	12.0(5)	[S21]
$CaWO_4$	4.94(2)	Direct	Γ	Γ	-2.1(3)	[S23]
$SrWO_4$	4.98(4)	Direct	Γ	Γ	3.7(7)	[S23]
$EuWO_4$	3.1(1)	Indirect	X	Γ	-42	[S16, S24, S25]
$PbWO_4$	4.01(2)	Indirect	Δ	Σ	-62(2)	[S23]
$BaWO_4$	5.20(3)	Direct	Γ	Γ	8.9(8)	[S23]

Table S2: Band-gap energy (E_{gap}) and pressure coefficient (dE_{gap}/dP) of different scheelite and wolframite tungstates; values taken from the literature.[S2, S3, S5, S16, S21, S22, S23, S24, S25] In the table we indicate whether the band gap is direct or indirect. We also provide the location within the Brillouin zone of the valence-band maximum (VBM) and conduction-band minimum (CBM).

Atom	x	y	z
W	0.23272	0.03194	0.16136
Be	0.20406	0.35219	0.54937
O1	0.32276	0.22402	0.33894
O2	0.38489	0.30963	0.81496
O3	0.12674	0.69980	0.42436
O4	0.10120	0.78988	0.94354

Table S3: Calculated fractional atomic coordinates of the triclinic structure of $BeWO_4$.

Mode	ω (cm^{-1})	Mode	ω (cm^{-1})
A_g	125.3	A_u	210.4
A_g	136.6	A_u	230.5
A_g	195.5	A_u	287.9
A_g	203.0	A_u	303.0
A_g	292.3	A_u	344.4
A_g	300.4	A_u	397.4
A_g	321.9	A_u	439.8
A_g	384.5	A_u	535.5
A_g	439.6	A_u	539.6
A_g	465.5	A_u	575.1
A_g	513.6	A_u	652.4
A_g	540.9	A_u	696.4
A_g	602.3	A_u	738.6
A_g	707.2	A_u	834.9
A_g	767.9	A_u	945.6
A_g	805.2		
A_g	847.1		
A_g	949.1		

Table S4: Calculated frequencies of the Raman-active (A_g) and IR-active (A_u) modes of $BeWO_4$.

Raman-active		Infrared-active	
**Mode**	**ω(P) (cm^{-1})**	**Mode**	**ω(P) (cm^{-1})**
B_g	$156 + 0.49\ P - 0.0088\ P^2$	A_u	$155 + 0.10\ P - 0.0098\ P^2$
A_g	$156 - 2.66\ P - 0.0465\ P^2$	B_u	$253 + 0.61\ P - 0.0245\ P^2$
B_g	$162 + 0.48\ P - 0.0065\ P^2$	B_u	$313 + 0.08\ P - 0.0066\ P^2$
A_g	$244 + 0.49\ P - 0.0282\ P^2$	B_u	$338 + 0.36\ P - 0.0118\ P^2$
A_g	$275 + 0.51\ P + 0.0004\ P^2$	A_u	$342 + 0.27\ P - 0.0045\ P^2$
B_g	$306 + 0.30\ P - 0.0100\ P^2$	B_u	$430 + 2.94\ P - 0.0468\ P^2$
A_g	$350 + 0.76\ P - 0.0197\ P^2$	A_u	$430 + 3.43\ P - 0.0190\ P^2$
A_g	$405 + 3.49\ P - 0.0341\ P^2$	B_u	$434 + 3.13\ P - 0.0090\ P^2$
B_g	$421 + 2.50\ P - 0.0089\ P^2$	B_u	$511 + 2.87\ P - 0.0153\ P^2$
A_g	$474 + 0.95\ P - 0.0067\ P^2$	A_u	$520 + 3.20\ P - 0.0193\ P^2$
B_g	$486 + 2.75\ P - 0.0143\ P^2$	A_u	$559 + 3.62\ P - 0.0122\ P^2$
B_g	$512 + 3.17\ P - 0.0212\ P^2$	B_u	$590 + 4.57\ P - 0.0171\ P^2$
A_g	$512 + 3.21\ P + 0.0001\ P^2$	B_u	$643 + 5.28\ P - 0.0186\ P^2$
B_g	$561 + 2.60\ P - 0.0100\ P^2$	A_u	$714 + 4.30\ P - 0.0184\ P^2$
A_g	$626 + 5.53\ P - 0.0280\ P^2$	B_u	$796 + 5.10\ P - 0.0199\ P^2$
A_g	$735 + 5.37\ P - 0.0208\ P^2$		
B_g	$754 + 4.14\ P - 0.0099\ P^2$		
A_g	$874 + 4.82\ P - 0.0236\ P^2$		

Table S5: Calculated pressure dependence of phonon frequencies for Raman- and infrared-active phonons. In the polynomial P is the pressure in GPa.

References

[S1] Vilaplana, R.; Lacomba-Perales, R.; Gomis, O.; Errandonea, D.; Meng, Y. Quasi-hydrostatic X-ray powder diffraction study of the low- and high-pressure phases of $CaWO_4$ up to 28 GPa. *Solid State Sci.* **2014**, *36*, 16-23, DOI: 10.1016/j.solidstatesciences.2014.07.003

[S2] Diaz-Anichtchenko, D.; Aviles-Coronado, J. E.; López-Moreno, S.; Turnbull, R.; Manjón, F. J.; Popescu, C.; Errandonea, D. Electronic, Vibrational, and Structural Properties of the Natural Mineral Ferberite ($FeWO_4$): A High-Pressure Study. *Inorg. Chem.* **2024**, *63*, 6898-6908, DOI: 10.1021/acs.inorgchem.4c00345

[S3] Errandonea, D.; Rodriguez, R.; Vilaplana, R.; Vie, D.; Garg, S.; Nayak, B.; Garg, N.; Singh, J.; Kanchana, V.; Vaitheeswaran. Band-Gap Energy and Electronic d–d Transitions of $NiWO_4$ Studied under High-Pressure Conditions. *J. Phys. Chem. C* **2023**, *127*, 15630–15640, DOI: 10.1021/acs.jpcc.3c03512

[S4] Ruiz-Fuertes, J.; López-Moreno, S.; Errandonea, D.; Pellicer-Porres, J.; Lacomba-Perales, R.; Segura, A.; Rodríguez-Hernández, P.; Muñoz, A.; Romero, A.H.; González, J. High-pressure phase transitions and compressibility of wolframite-type tungstates. *J. Appl. Phys.* **2010**, *107*, 083506, DOI: 10.1063/1.3380848

[S5] Bandiello, E.; Rodríguez-Hernández, P.; Muñoz, A.; Bajo Buenestado, M.; Popescu, C.; Errandonea, D. Electronic properties and high-pressure behavior of wolframite-type $CoWO_4$. *Mater. Adv.* **2021**, *2*, 5955-5966, DOI: 10.1039/D1MA00510C

[S6] Ruiz-Fuertes, J.; Friedrich, A.; Pellicer-Porres, J.; Errandonea, D.; Segura, A.; Morgenroth, W.; Haussühl, E.; Tu, C. T.; Polian, A. Structure Solution of the High-Pressure Phase of $CuWO_4$ and Evolution of the Jahn–Teller Distortion. *Chem. Mat.* **2011**, *23*, 4220-4226, DOI: 10.1021/cm201592h

[S7] Ruiz-Fuertes, J.; Errandonea, D.; Lacomba-Perales, R.; Segura, A.; González, J.; Rodríguez, F.; Manjón, F. J.; Ray, S.; Rodríguez-Hernández, P.; Muñoz, A.; Zhu, Z.; Tu, C. Y. High-pressure structural phase transitions in $CuWO_4$. *Phys. Rev. B* **2010**, *81*, 224115, DOI: 10.1103/PhysRevB.81.224115

[S8] Ruiz-Fuertes, J.; Friedrich, A.; Gomis, O.; Errandonea, D.; Morgenroth, W.; Sans, J.A.; Santamaría-Pérez, D. High-pressure structural phase transition in $MnWO_4$. *Phys. Rev. B* **2015**, *91*, 104109, DOI: 10.1103/PhysRevB.91.104109

[S9] Ruiz-Fuertes, J.; Friedrich, A.; Errandonea, D.; Segura, A.; Morgenroth, W.; Rodríguez-Hernández, P.; Muñoz, A.; Meng, Y. Optical and structural study of the pressure-induced phase transition of $CdWO_4$. *Phys. Rev. B* **2017**, *95*, 174105, DOI: 10.1103/PhysRevB.95.174105

[S10] Hazen, R. M.; Finger, L. W.; Mariathasan, J. W. E.; High-pressure crystal chemistry of scheelite-type tungstates and molybdates. *J. Phys. Chem. Sol.* **1985**, *253-263*, DOI: 10.1016/0022-3697(85)90039-3.

[S11] Grzechnik, A.; Crichton, W. A.; Hanfland, M., Van Smaalen, S. Scheelite $CaWO_4$ at high pressures. *J. Phys. Condens. Matter* **2003**, *15*, 7261, DOI 10.1088/0953-8984/15/43/010

[S12] Errandonea, D.; Pellicer-Porres, J.; Manjon, F. J.; Segura, A.; Ferrer-Roca, Ch.; Kumar, R. S.; Tschauner, O.; Rodríguez-Hernández, P.; López-Solano, J.; Radescu, S.; Mujica, A.; Muñoz, A.; Aquilanti. G. High-pressure structural study of the scheelite tungstates $CaWO_4$ and $SrWO_4$. *Phys. Rev. B* **2005**, *72*, 174106, DOI: 10.1103/PhysRevB.72.174106

[S13] Errandonea, D.; Somayazulu, M.; Häusermann, D. Phase transitions and amorphization of $CaWO_4$ at high pressure. *Phys. Stat. Sol. B* **2003**, *235*, 162-169, DOI: 10.1002/pssb.2003014124

[S14] Manjón, F. J.; López-Solano, J.; Ray, S.; Gomis, O.; Santamaría-Pérez, D.; Mollar, M.;

Panchal, V.; Errandonea, D.; Rodríguez-Hernández, P.; Muñoz, A.; High-pressure structural and lattice dynamical study of $HgWO_4$. *Phys. Rev. B* **2010**, *82*, 035212, DOI: 10.1103/PhysRevB.82.035212

[S15] Diaz-Anichtchenko, D.; Ibáñez, J.; Botella, P., Oliva, R.; Kuzmin, A.; Wang, L.; Li, Y.; Muñoz, A.; Alabarse, F.; Errandonea, D.; Identification of the high-pressure phases of a-$SnWO_4$ combining x-ray diffraction and crystal structure prediction. *Physica B* **2025**, *696*, 416666, DOI: 10.1016/j.physb.2024.416666

[S16] Errandonea, D. High-pressure X-ray diffraction study of $EuWO_4$ to 12 GPa. *Phys. Stat. Sol. B* **2005**, *242*, R125-R127, DOI: 10.1002/pssb.200541334

[S17] Errandonea, D.; Pellicer-Porres, J.; Manjon, F. J.; Segura, A.; Ferrer-Roca, Ch.; Kumar, R. S.; Tschauner, O.; Rodríguez-Hernández, P.; López-Solano, J.; Radescu, S.; Mujica, A.; Muñoz, A.; Aquilanti. G. Determination of the high-pressure crystal structure of $BaWO_4$ and $PbWO_4$. *Phys. Rev. B* **2006**, *73*, 224103, DOI: 10.1103/PhysRevB.73.224103

[S18] Panchal, V.; Garg, N.; Chauhan, A. K.; Sangeeta, Sharma, S. M. High pressure phase transitions in $BaWO_4$. *Solid State Commun.* **2004**, *130*, 203-208, DOI: 10.1016/j.ssc.2004.01.043.

[S19] Ashraf, R.; Shehzadi, Z.; Mahmood, T.; Naeem, S.; Shehzadi, N.; Iftikhar, S.; Parveen, Z. DFT based investigations of $BaWO_4$: Electronic and optical properties. *Physica B* **2012**, *621*, 413309, DOI: 10.1016/j.physb.2021.413309.

[S20] Ye, M.; Zhou, Y.; Shao, T.; Liu, H.; Tao, Q.; Wang, X.; Tang, R.; Yue, H.; Li, Y.; Zhu, P. Effects of High Pressure on the Bandgap and the d–d Crystal Field Transitions in Wolframite $NiWO_4$. *J. Phys. Chem. C* **2023**, *127*, 6543-6551. DOI: 10.1021/acs.jpcc.2c09036

[S21] Ruiz-Fuertes, J.; López-Moreno, S.; López-Solano, J.; Errandonea, D.; Segura, A.; Lacomba-Perales, R.; Muñoz, A.; Radescu, S.; Rodríguez-Hernández, P.; Gospodinov, M.; Nagornaya, L. L.; Tu, C. Y. Pressure effects on the electronic and optical properties of AWO_4 wolframites (A = Cd, Mg, Mn, and Zn): The distinctive behavior of multiferroic $MnWO_4$. *Phys. Rev. B* **2012**, *86*, 125202, DOI: 10.1103/PhysRevB.86.125202

[S23] Ruiz-Fuertes, J.; Errandonea, D.; Segura, A.; Manjón, F. J.; Zhu, Z.; Tu, C. Y. Growth, characterization, and high-pressure optical studies of $CuWO_4$. *High Pres. Res.* **2008**, *28*, 565-570, DOI: 10.1080/08957950802446643

[S23] Lacomba-Perales; R.; Errandonea; D.; Segura; A.; Ruiz-Fuertes; J.; Rodríguez-Hernández; P.; Radescu; S.; López-Solano; J.; Mujica; A.; Muñoz, A. A combined high-pressure experimental and theoretical study of the electronic band-structure of scheelite-type AWO_4 (A = Ca, Sr, Ba, Pb) compounds. *J. Appl. Phys.* **2011**, *110*, 043703, DOI: 10.1063/1.3622322

[S24] Lal, H. B.; Dar, N.; Kumar, A. On the electrical conductivity, dielectric constant and magnetic susceptibility of $EuWO_4$. J. Phys. C 1974, 7, 4335-4345, DOI: 10.1088/0022-3719/7/23/020

[S25] Zi, Y.; Huang, A.; Zhao, H.; Bai, X.; Liu, Y.; Cun, Y.; Song, Z.; Qiu, J.; Shen, Y.; Zhou, J.; Yang, Z. Force-light-heat stimulation-induced multicolor chromism and multifunctional applications of europium tungstate phosphor. Nat. Commun. 2025, 16, 6857. DOI: 10.1038/s41467-025-62167-1
